# A predictive model for flow index performance of pit drip irrigation emitters using BP-PSO algorithm

**DOI:** 10.1038/s41598-026-56406-8

**Published:** 2026-06-04

**Authors:** Weixiong Xu, Jiatong Jiang, Tianyu Xu

**Affiliations:** 1 School of Hydraulic and Electric Power, Heilongjiang University, Harbin, 150080 China; 2 Engineering Research Center of Agricultural Microbiology Technology, Ministry of Education, Heilongjiang University, Harbin, 150500 China

**Keywords:** Plant sciences, Engineering

## Abstract

It is crucial to accurately obtain the flow index in designing and developing labyrinth drip irrigation emitters. This study designed a pit drip irrigation emitter based on plant biomimetic principles, and created training-testing datasets (160 data points) and external validation datasets (25 data points) through experiments. The improved backpropagation neural network (BP) regression algorithm based on particle swarm optimization (PSO) is used to predict the flow index of the pit drip irrigation emitter. The input parameters include the pit depth *l*, the inner and outer boundary spacing *h*, the flow channel angle *θ*, and the pit aperture *j*. There were four combinations with the different hidden layer settings, namely BP_1_, BP_2_, BP_1_-PSO, BP_2_-PSO. 85% of the training-testing dataset was used to train the developed model (using 5-fold cross-validation), of which 15% was used for testing. The reliability of the prediction model was evaluated using mean absolute error (MAE), mean square error (MSE), root mean square error (RMSE), mean absolute percentage error (MAPE), uncertainty at 95%(U_95_), coefficient of determination (R^2^), and global performance indicator (GPI). The results indicated that the optimal structures of BP_1_ and BP (input-hidden layer-output) were 4-7-1 and 4-4-9-1. The BP_2_-PSO model had the best prediction accuracy and stability, MAE, MSE, RMSE, MAPE, U95, R², and GPI values were 1.98 × 10^− 3^, 5.49 × 10^− 6^, 2.34 × 10^− 3^, 4.09 × 10^− 3^, 6.46 × 10^− 3^, 0.9456, and 3.74 × 10^− 3^, respectively. the evaluation values were 6.46 × 10^− 3^, 0.9456 and 3.74 × 10^− 3^ respectively. The GPI of BP_1_, BP_2_, BP_1_-PSO and BP_2_-PSO were 6.35 × 10^− 3^, 5.96 × 10^− 3^, 4.88 × 10^− 3^ and 3.74 × 10^− 3^ respectively, indicating good prediction accuracy. The ranking of prediction accuracy was as follows: BP_2_-PSO > BP_1_-PSO > BP_2_ > BP_1_. The four models were tested on the external validation dataset, the R² values of all four models were higher than 0.85, indicating good correlation. Through SHAP analysis, the importance of structural parameters to the flow index is ranked as: *j* > *θ* > *h* > *l*. In order to verify the validity and extensive superiority of BP-PSO model in solving drip irrigation emitter flow index prediction problem, it was compared with six representative machine learning models, including classical models (GR and MLR), integrated models (CatBoost and LSBoost) and hybrid models (MLP-SVM and MLP-DT). The BP_2_-PSO and BP_1_-PSO models had the highest prediction accuracy in the training-testing process among ten machine learning methods, and BP_2_-PSO model had the highest prediction accuracy in the external validation process, which indicated that particle swarm optimization can improve the accuracy of neural network models. The BP_2_-PSO model was used to predict the flow index had greater advantages during the design and development stages of pit drip irrigation emitters.

## Introduction

The rational utilization of water resources is an important measure to promote agricultural development^1–3^. Drip irrigation is core technology in the field of water-saving irrigation, it has been widely applied in global agricultural water management^4^. The performance of drip irrigation emitters can reflect the working efficiency of the drip irrigation system^5,6^. The flow index is an important indicator for measuring the hydraulic performance of drip irrigation emitters, and its value ranges be-tween 0 and 1^7^. The smaller the flow index has the lower the sensitivity of drip irrigation emitter to the working pressure^8^. The structure of the flow channel can lead to some differences in the flow index. It is crucial to accurately and quickly establish the relationship between the flow channel structure parameters and the flow index during the design, development, and optimization stages.

At present, most scholars adopted single-factor or multi-factor orthogonal experiments and genetic algorithms to explore the relationship between the flow channel structure parameters and the flow index. ZHANG et al.^9^ employed the single-factor experimental method to explore the impact of the inter-tooth angle of the labyrinth flow channel on the hydraulic performance of drip irrigation emitters. The results showed the inter tooth angle increased, the flow quantity inside the drip irrigation emitter increased. When the inter tooth angle was 70°, the hydraulic performance was optimal. Single factor experiments had certain advantages for preliminary exploration and simple causal relationship analysis, but multi factor experiments is crucial to study of complex systems. Hou et al.^10^ utilized orthogonal experiments to study the influence of flow channel geometric parameters on hydraulic performance, and obtained the equation of the relationship between the flow index and these parameters through multiple linear regression analysis. The advantage of multi-factor experiments was that they can explore and explain the behavior of complex systems more comprehensively. However, the complexity of such experimental designs and the difficulty of data analysis were relatively high. Hu et al.^11^ designed the structural parameters of the flow channel using genetic algorithms and analyzed its hydraulic performance using CFD tool. The result shown through regression equations that the tooth staggering value was the most important factor affecting hydraulic performance. The genetic algorithm can provide effective optimization solutions in solving complex, nonlinear, nonconvex, combinatorial optimization and other problems. Its disadvantage was prone to prematurely converge to a local optimal solution, failing to find the global optimal solution.

The above experiment combined with regression analysis model indirectly obtained the relationship between flow index and flow channel structural parameters. This method only discussed the independent influence of a single structural parameter. It was important to consider the interaction effects among the structural parameters^12,13^. Developing more reasonable, simple, and direct prediction methods was crucial for the design and development of drip irrigation emitters. When it comes to the multi-objective optimization, non-linearity and prediction problems faced in the de-sign of drip irrigation emitters, the use of machine learning methods has significant advantages^14–16^. Guo et al.^17^ developed a flow quantity prediction model for drip irrigation emitters by using support vector machine (SVM). The average error between this model and the experimental samples was 2.25%, demonstrating the high accuracy of the model. Mattar et al.^18^ employed the artificial neural network (ANN) model and gene expression programming (GEP) model to predict the flow quantity variations of drip irrigation emitters. They found that the ANN model had a lower error compared with the GEP model, indicating that the ANN model had a significant advantage in predicting the hydraulic performance of drip irrigation emitters. Chen et al.^19^ predicted the flow index of labyrinth drip irrigation emitters by using the response surface method (RSM) in combination with the ANN and support vector regression (SVR) models. The results showed that the SVR model had the highest prediction accuracy. The results showed that the SVR model had the highest prediction accuracy. Li et al.^20^ developed extreme learning machine (ELM), backpropagation neural network (BPNN) and traditional multiple linear regression (MLR) models to predict the flow index of SWRLC drip irrigation emitters. The comparison results showed that the ELM model performed the best, followed by the BPNN model. Machine learning method had achieved good results in the hydraulic performance prediction of drip irrigation emitters. The single model prediction method had limitations. There were many parameters or the nonlinear degree is high, the prediction results will be difficult to obtain the optimal solution. In hybrid algorithms, Achite et al.^21^ compared the predictive performance of standard SVR against two novel hybrid models (HBA-SVR and COOT-SVR) for monthly pan evaporation prediction in Algeria. Results demonstrated that employing the COOT optimization algorithm to identify optimal SVR parameter configurations significantly enhanced model performance when addressing complex prediction tasks involving multivariate, strongly non-linear relationships. Samantaray et al.^22^ pioneered the integration of a novel Sparrow Search Algorithm into SSL for optimizing SVM hyperparameters, thereby constructing an SVM-SSA hybrid forecasting model. Research demonstrated that the SSA algorithm effectively optimizes SVM parameters, enhancing its performance in complex non-linear forecasting tasks. Mirzania et al.^23^ proposed a novel hybrid model (AIG-SVR) for forecasting monthly water level variations in Lake Michigan and Lake Superior within the Great Lakes of North America. Results demonstrated that compared to a standalone SVR model, AIG-SVR exhibited superior generalization capabilities, successfully enhancing prediction accuracy. This provides water resource managers with a reliable tool to support decision-making.

Combining different machine learning methods with optimization models (hybrid algorithms) is more conducive to model prediction. BP neural networks have non-linear fitting, self-learning and generalization capabilities. In order to predict the flow index of perforated drip irrigation emitters, the best hidden layer of the BP model is determined through five-fold cross verification to improve the prediction accuracy. Most of the existing hybrid model research focuses on internal verification and comparison, and lacks strict testing of its generalization ability and the applicability of engineering algorithms. This study conducts external verification based on the generalization ability of the algorithm to obtain the best flow index prediction accuracy. PSO is a population optimization algorithm that uses “particles” to simulate each bird in the flock and optimize the thresholds and input weights of the BP neural network. It has certain advantages in terms of model stability, convergence accuracy and speed^24,25^. Six different machine learning algorithms (Categorical boosting (CatBoost), Least squares boosting (LSBoost), Multilayer perceptron-support vector machine (MLP-SVM), Multi-layer perceptron-determination tree (MLP-DT), Gaussian regression (GR), and Multiple linear regression (MLR))) were simultaneously compared to determine the overall performance of the BP-PSO model. The purpose of this study was to: (1) construct a PSO-BP hybrid structure to solve the problem that BP neural network is sensitive to initial weights and easily falls into local optimality; (2) utilize external validation methods to assess the actual generalization ability of the model in future applications and employ SHAP analysis to evaluate the importance of structural parameters to the flow index; (3) design multi-class model comparison experiments to verify the versatility of BP-PSO. This study provides data and theoretical support for the design and performance optimization of drip irrigation emitters.

## Materials and methods

**Fig. 1:**
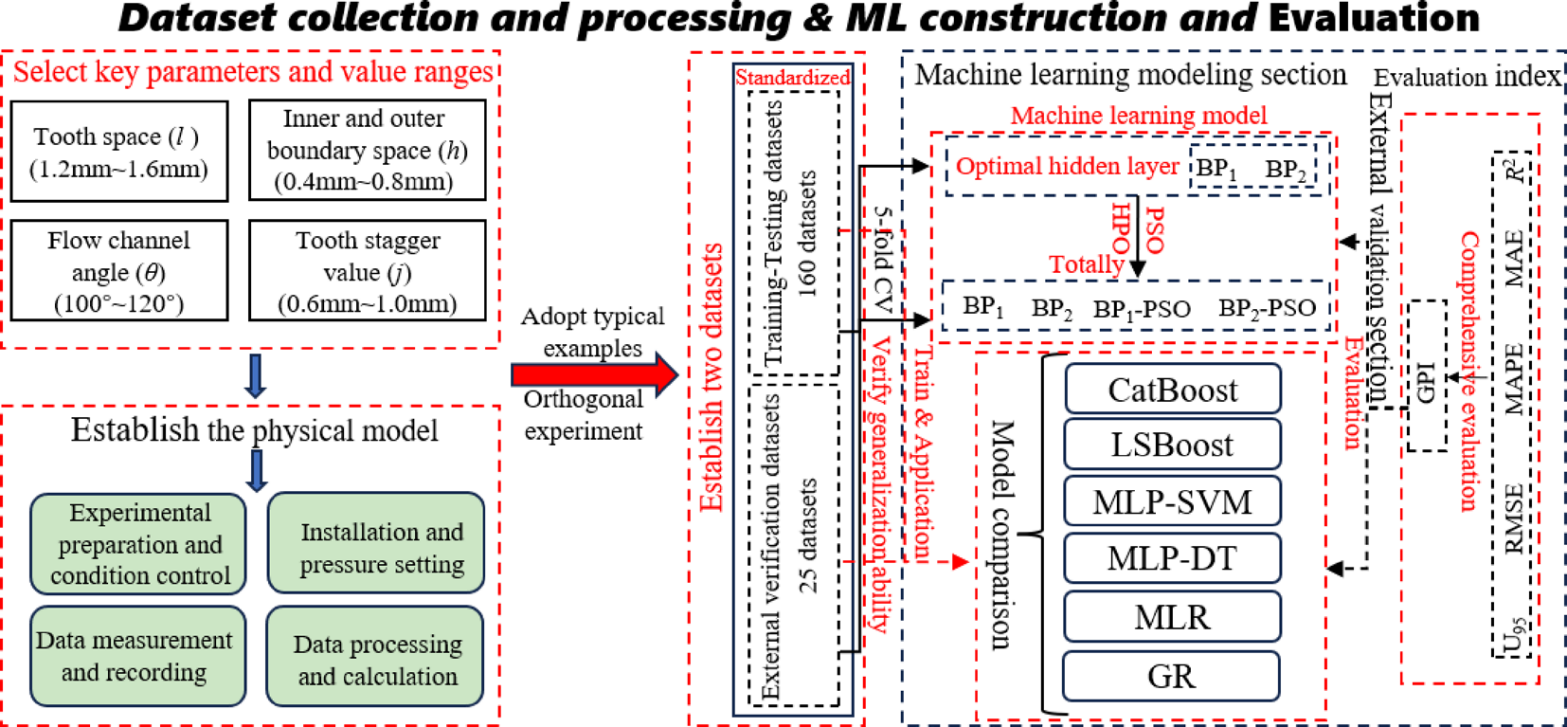
Technical roadmap diagram.

The flow index is one of the main indicators for measuring the hydraulic performance of drip irrigation emitters, and it is an important parameter that must be considered during the design and development stages of drip irrigation emitters. Predicting the flow index of drip irrigation emitters quickly and accurately is of great significance for optimizing the design of drip irrigation systems, improving irrigation uniformity and enhancing anti-clogging performance. In this study, Machine learning (ML) method techniques were adopted to establish the non-linear relationship between the flow channel structural parameters of pit emitters and its flow index. The research framework of the proposed model is shown in Fig. 1.

The research framework is divided into two parts. The first part is to screen four key structural parameters that affect the performance of drip irrigation emitters and determine their range of values. Subsequently, a controllable hydraulic performance test was carried out in the hydraulic laboratory to measure the stable flow of the drip irrigation emitter under different working pressures. Finally, the flow index was calculated through mathematical model (Eq. 1). Based on a large amount of experimental data, a mapping relationship data set of “Drip emitter structure parameters-Flow index” is constructed, and is divided into a training-testing set for training and adjusting models and an external verification set for final inspection. The research goes from simple to complex, and BP neural networks with single hidden layers (BP_1_) and dual hidden layers (BP_2_) are constructed as the basic models. PSO is introduced to intelligently optimize the hyperparameters of BP network, and two optimization models, BP_1_-PSO and BP_2_-PSO, are obtained. Six evaluation indicators are also introduced to make the evaluation very systematic. The optimized BP-PSO model is compared with six classic machine learning models such as CatBoost and LSBoost.

### Structural design and physical model of drip irrigation emitter

**Fig. 2:**
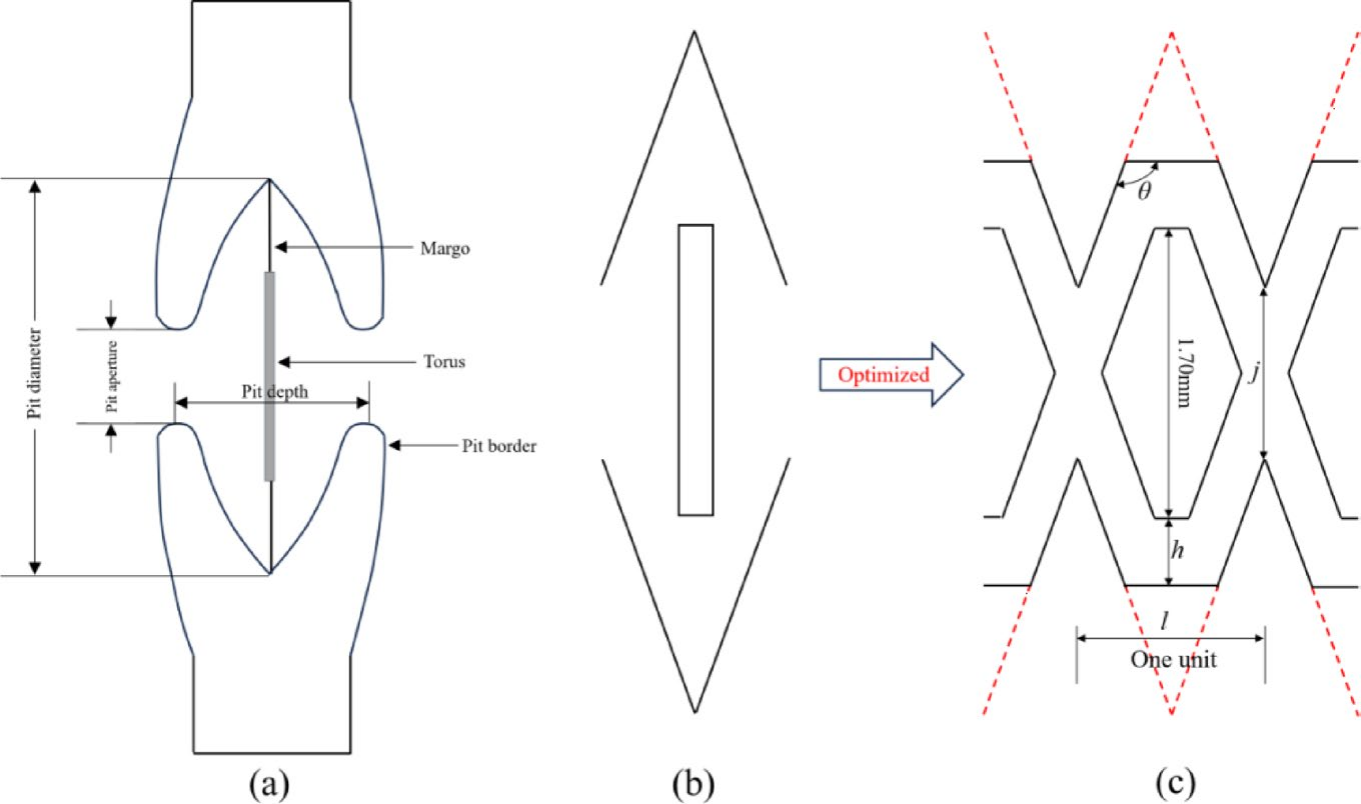
**(a)** Schematic of torus-margo border pit **(b)** PDIE model **(c)** Optimized PDIE model.

A new type of pit drip irrigation device with diversion, rapid rotation and con-vergence was constructed using plant biomimetic principles. Tracheids are one of the main structures for water transportation in the xylem of plants^26^. The transportation of water mainly relies on the pit structure between adjacent tracheids, and the pit structure has a stable pressure-drop effect. This study adopted the most representative torus margo structure, as shown in Fig. 2 (a), and designed a novel pit drip irrigation emitter (PDIE), as shown in Fig. 2 (b). The optimized design was achieved by reducing the flow area at the upper and lower ends of the PDIE and adding resistance-forming triangles on both sides of the torus margin (where the oblique edges of the inner ring of the flow channel were parallel to the adjacent inner tooth edges), as shown in Fig. 2(c)^27^.

**Fig. 3:**
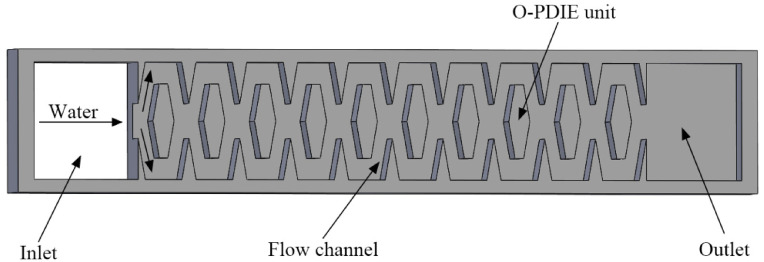
Schematic diagram o f O-PDIE physical model structure.

The pit flow channel consisted of four structural parameters. *l* represented the pit depth, *h* represented the inner and outer boundary spacing, *θ* represented the flow channel angle, and *j* represented the pit aperture. The flow channel depth D was set to 0.6 mm, the height of the inner ring of the flow channel was 1.70 mm, and the number of pit units was 10. The physical model is shown in Fig. 3. According to the actual design requirements of the drip irrigation emitter and reference^28^, the value ranges of the key geometric parameters were determined (Table 1).

**Table 1:** The value ranges of structural parameters of O-PDIE emitter.

Parameters	l(mm)	h(mm)	θ(°)	j(mm)
Ranges	1.2 ~ 1.6	0.4 ~ 0.8	100 ~ 120	0.6 ~ 1.0

### Experimental method

**Fig. 4:**
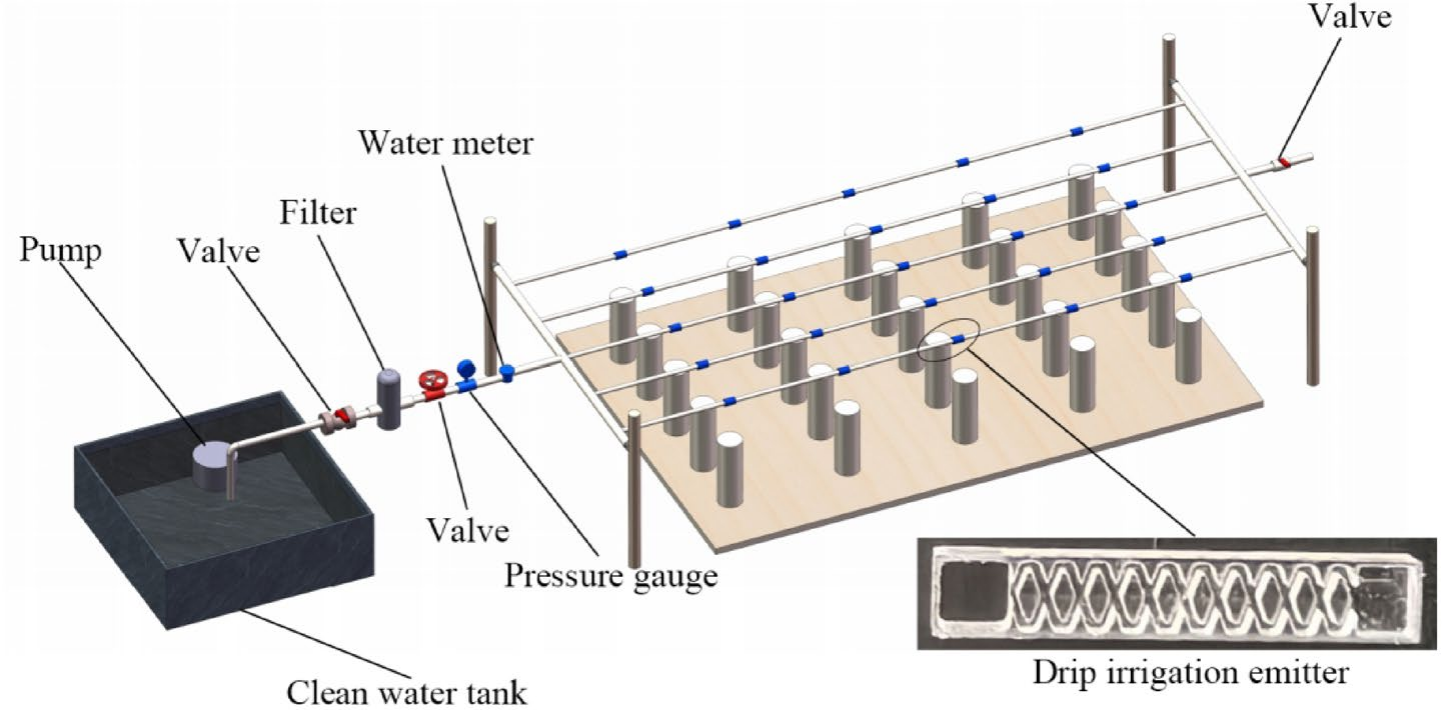
Physical and experimental setup of perforated drip irrigation emitter.

This study used 3D software to design a experimental model, which was carved using an EM-G32S-X32 high-precision carving machine (with a carving accuracy of 0.01 mm), and made of organic glass to form proportional pit drip irrigation emitters. Figure 4 showed the physical sample of the pit drip irrigation emitter and the experimental device. The measurement site is the School of Hydraulic and Electric Power of Heilongjiang University, the water temperature is 22 ~ 24℃, and the laboratory is a hydraulic branch room. The device mainly consisted of a water tank, a water pump, a pressure-reducing valve, a pressure gauge (0.1-grade precision), and several pipelines. The experimental device was divided into one layer, with five drip irrigation pipes installed on each layer. Each pipeline was equipped with 5 pit drip irrigation emitters, for a total of 25 pit drip irrigation emitters. Firstly, tested the flow quantity of the drip irrigation device with clean water under a working pressure of 50 kPa. Each test lasted for 5 min and was repeated 3 times. Recorded the flow quantity of each test with a measuring cylinder and took the average as the rated flow quantity. In the subsequent testing, the inlet pressure of the clean water experiment increased every 25 kPa and stopped at 250 kPa. The above operation was repeated to obtain the flow quantity at different working pressures.

The relationship between the pressure and flow quantity of the irrigation flow channel can be obtained^29^:

1 $$q={k_{\mathrm{d}}}{h^x}$$

In the formula, *q* is the flow rate value (L/h) of the irrigation emitter under a given pressure, *k*_d_ is the flow coefficient, *h* is the inlet pressure (kPa) and *x* is the flow index (whose value depends on the flow state inside the irrigation emitter).

The pressure and flow curves of the irrigation emitter was fitted through regression analysis using IBM SPSS Statistics 25 software, and the value of the flow index was obtained.

### Establishment and division of datasets

In this study, a total of two datasets were created. The first dataset contained 160 sets of experimental calculation values, excluding data points from orthogonal experiments. This dataset was only used for training and testing ML models and was named the training-testing dataset. The training-testing dataset was used to establish the ML model, as shown in Table 2. The second dataset was orthogonal experimental data points, which aims to analyze the performance of the trained ML model through external validation. The second dataset was named the external validation dataset, as shown in Table 3.

**Table 2:** Training-testing dataset.

Level	Geometry parameters values				Level	Geometry parameters values			
	l(mm)	h(mm)	θ(°)	j(mm)		l(mm)	h(mm)	θ(°)	j(mm)
1	1.2	0.4	105	0.8	81	1.4	0.4	110	1
2	1.2	0.4	110	1	82	1.4	0.5	105	0.7
3	1.2	0.4	115	0.7	83	1.4	0.5	110	0.9
4	1.2	0.5	100	1	84	1.4	0.5	115	0.6
5	1.2	0.5	105	0.7	85	1.4	0.6	100	0.9
6	1.2	0.6	100	0.9	86	1.4	0.6	105	0.6
7	1.2	0.6	110	0.8	87	1.4	0.6	115	0.7
8	1.2	0.6	115	1	88	1.4	0.7	100	0.8
9	1.2	0.7	100	0.8	89	1.4	0.7	105	1
10	1.2	0.7	110	0.7	90	1.4	0.7	110	0.7
11	1.2	0.7	115	0.9	91	1.4	0.7	115	0.9
12	1.2	0.8	100	0.7	92	1.4	0.8	100	0.7
13	1.2	0.8	105	0.9	93	1.4	0.8	110	0.6
14	1.2	0.8	110	0.6	94	1.4	0.8	115	0.8
15	1.2	0.8	115	1	95	1.4	0.4	110	0.6
16	1.2	0.6	100	1	96	1.4	0.4	115	0.8
17	1.2	0.5	100	0.7	97	1.4	0.4	115	0.9
18	1.2	0.6	100	1	98	1.4	0.4	120	0.9
19	1.2	0.6	105	0.7	99	1.4	0.5	120	0.8
20	1.2	0.4	100	0.7	100	1.4	0.6	105	0.6
21	1.2	0.4	100	0.8	101	1.4	0.6	120	1
22	1.2	0.4	100	0.9	102	1.4	0.7	120	0.8
23	1.2	0.4	100	1	103	1.4	0.8	105	0.9
24	1.2	0.4	105	0.6	104	1.4	0.8	115	0.8
25	1.2	0.4	105	0.7	105	1.4	0.8	120	1
26	1.2	0.4	105	0.9	106	1.4	0.4	120	0.9
27	1.2	0.4	105	1	107	1.4	0.6	120	1
28	1.2	0.4	110	0.6	108	1.5	0.4	100	0.6
29	1.2	0.4	110	0.7	109	1.5	0.4	105	1
30	1.2	0.4	115	0.8	110	1.5	0.4	110	0.9
31	1.2	0.4	115	0.9	111	1.5	0.4	115	0.8
32	1.2	0.4	115	1	112	1.5	0.5	100	0.8
33	1.2	0.5	100	0.7	113	1.5	0.5	105	0.7
34	1.2	0.5	100	0.8	114	1.5	0.5	110	0.6
35	1.2	0.6	110	0.6	115	1.5	0.5	115	1
36	1.2	0.6	110	0.7	116	1.5	0.6	100	1
37	1.2	0.6	110	0.9	117	1.5	0.6	105	0.9
38	1.2	0.6	110	1	118	1.5	0.6	110	0.8
39	1.2	0.6	100	0.7	119	1.5	0.6	115	0.7
40	1.2	0.6	100	0.8	120	1.5	0.7	100	0.7
41	1.2	0.6	100	1	121	1.5	0.7	105	0.6
42	1.2	0.5	100	0.9	122	1.5	0.7	110	1
43	1.2	0.6	105	0.7	123	1.5	0.8	100	0.9
44	1.2	0.6	105	0.8	124	1.5	0.8	105	0.8
45	1.2	0.6	105	0.9	125	1.5	0.8	110	1
46	1.2	0.5	110	0.7	126	1.5	0.8	115	0.6
47	1.2	0.5	110	0.8	127	1.5	0.4	115	0.7
48	1.2	0.5	105	0.9	128	1.5	0.5	105	0.6
49	1.2	0.5	115	0.6	129	1.5	0.5	105	0.8
50	1.2	0.6	105	0.6	130	1.5	0.5	105	0.9
51	1.2	0.7	105	1	131	1.5	0.5	120	0.9
52	1.3	0.4	100	0.6	132	1.5	0.7	115	0.9
53	1.3	0.4	105	0.9	133	1.5	0.7	120	0.8
54	1.3	0.4	110	0.7	134	1.5	0.8	110	0.7
55	1.3	0.4	115	1	135	1.5	0.4	120	0.7
56	1.3	0.5	100	0.9	136	1.6	0.7	120	0.6
57	1.3	0.5	110	1	137	1.6	0.4	110	1
58	1.3	0.5	115	0.8	138	1.6	0.4	120	0.9
59	1.3	0.6	100	0.7	139	1.6	0.5	120	0.8
60	1.3	0.6	105	1	140	1.6	0.6	120	0.7
61	1.3	0.6	110	0.8	141	1.6	0.8	120	0.8
62	1.3	0.6	115	0.6	142	1.6	0.4	100	0.6
63	1.3	0.7	105	1	143	1.6	0.4	115	0.7
64	1.3	0.7	110	0.6	144	1.6	0.5	100	1
65	1.3	0.7	115	0.7	145	1.6	0.5	105	0.7
66	1.3	0.8	100	0.8	146	1.6	0.5	110	0.9
67	1.3	0.8	105	0.6	147	1.6	0.6	105	0.6
68	1.3	0.8	110	0.7	148	1.6	0.6	110	0.8
69	1.3	0.8	115	1	149	1.6	0.6	115	1
70	1.3	0.6	110	1	150	1.6	0.7	100	0.8
71	1.3	0.7	105	0.7	151	1.6	0.7	105	1
72	1.3	0.5	110	0.8	152	1.6	0.7	115	0.9
73	1.3	0.5	110	0.9	153	1.6	0.8	100	0.7
74	1.3	0.5	105	0.7	154	1.6	0.8	105	0.9
75	1.3	0.4	120	0.8	155	1.6	0.8	110	0.6
76	1.3	0.7	120	0.9	156	1.6	0.8	115	1
77	1.3	0.6	120	0.9	157	1.6	0.8	100	0.8
78	1.3	0.7	100	0.8	158	1.6	0.6	105	1
79	1.4	0.4	100	0.6	159	1.6	0.5	110	1
80	1.4	0.4	105	0.8	160	1.6	0.5	115	0.7

Both datasets consisted of 4 inputs and 1 output. Determine key geometric parameters according to relevant requirements and selected variables for input and output. The pit depth *l*, the inner and outer boundary spacing *h*, the flow channel angle *θ*, and the pit aperture *j* were used as the input parameters of the prediction model, while the flow index *x* was the output parameter employed in this study.

**Table 3:** External validation dataset.

Level	Geometry parameters values				Level	Geometry parameters values			
	l(mm)	h(mm)	θ(°)	j(mm)		l(mm)	h(mm)	θ(°)	j(mm)
1	1.2	0.4	100	0.6	14	1.4	0.7	120	0.6
2	1.2	0.5	110	0.9	15	1.4	0.8	105	0.9
3	1.2	0.6	120	0.7	16	1.5	0.4	110	1
4	1.2	0.7	105	1	17	1.5	0.5	120	0.8
5	1.2	0.8	115	0.8	18	1.5	0.6	105	0.6
6	1.3	0.4	120	0.9	19	1.5	0.7	115	0.9
7	1.3	0.5	105	0.7	20	1.5	0.8	100	0.7
8	1.3	0.6	115	1	21	1.6	0.4	105	0.8
9	1.3	0.7	100	0.8	22	1.6	0.5	115	0.6
10	1.3	0.8	110	0.6	23	1.6	0.6	100	0.9
11	1.4	0.4	115	0.7	24	1.6	0.7	110	0.7
12	1.4	0.5	100	1	25	1.6	0.8	120	1
13	1.4	0.6	110	0.8					

**Fig. 5:**
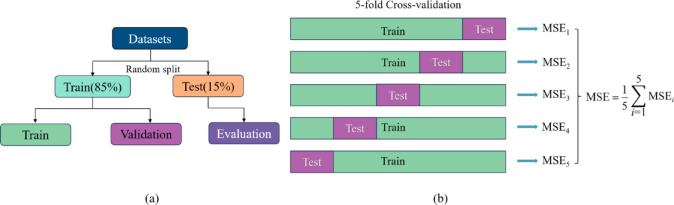
**(a)**. experimental data division and **(b)**. K-fold cross-validation.

The training-testing dataset was randomly shuffled. 85% of the data was used as the training subset, while the remaining 15% served as the testing subset. Determine the validation set in the training subset using the k-fold cross validation method (k = 5). During the training process, the particle swarm optimization algorithm and k-fold CV were utilized to adjust the hyperparameters. After hyperparameter optimization, the testing subset was used to evaluate the model. K-fold CV randomly divided the original training subset into k sub-datasets. Among them, k-1 sub-datasets were used as the training set, and the remaining dataset was used as the new testing set. This process was repeated 5 times, and the performance of the model was evaluated by calculating the average mean squared error (MSE) of the testing sets in the 5 sub-datasets. In this way, all available data participated in the training and testing phases. The specific process was shown in Fig. 5.

### Establishment of flow index prediction model

The BP is an artificial neural network based on the error back-propagation algorithm. It consists of three parts: the input layer, the hidden layer, and the output layer. The hidden layer can contain multiple neurons. The pit depth *l*, the inner and outer boundary spacing *h*, the flow channel angle *θ*, and the pit aperture *j* were the input values, and the flow index *x* was the predicted output value. The structure of the pit drip irrigation emitter was determined, the network was trained using the input and output datasets. the network parameters (weights and thresholds) were continuously adjusted to enable the network to achieve or approximate the desired input-output mapping relationship by back-propagating the error and simultaneously correcting the error.

The specific steps are as follows:

Step 1: Initialization of the network. Assume that the number of nodes in the input layer was *n*, the number of nodes in the hidden layer was *n*_1_, and the number of nodes in the output layer was *m*. The weight from the input layer to the hidden layer was *w*_ij_, the weight from the hidden layer to the output layer was *w*_jk_, the bias from the input layer to the hidden layer was *a*_j_, the bias from the hidden layer to the output layer was *b*_k_, and the learning rate was *η*.

Step 2: Output of the hidden layer. The output *H*_j_ of the hidden layer was:

2 $${H_j}=g\left( {\sum\limits_{{i=1}}^{n} {{w_{ij}}{x_i} - {a_j}} } \right)$$

Step 3: The output of the output layer. The output Ok of the output layer was:

3 $${O_k}=\sum\limits_{{j=1}}^{{{n_1}}} {({H_j}} {w_{jk}}) - {b_k}$$

Step 4: The calculation of the error. The error value *E* was:

4 $$E = {Y_k} - {O_k}$$

In the formula, *Y*_*k*_ represented the actual value, and *O*_*k*_ represented the predicted value.

Step 5: Weight update. In the process of error back-propagation, the gradient descent method was used to update the weights. The gradient direction was determined by calculating the partial derivatives of the loss function with respect to the weights. The weights from the hidden layer to the output layer *w*ij were updated, and the weights from the input layer to the hidden layer *w*_jk_ were updated. The weight update formulas were as follows:

5 $$\left\{ {\begin{array}{*{20}{c}} {{w_{ij}} = {w_{ij}} + \eta {H_j}\left( {1 - {H_j}} \right){x_i}\sum\limits_{k = 1}^m {{w_{jk}}{e_k}} }\\ {{w_{jk}} = {w_{jk}} + \eta {H_j}{e_k}} \end{array}} \right.$$

Step 6: Bias update. The bias from the input layer to the hidden layer aj was updated, and the bias from the hidden layer to the output layer bk was updated. The bias update formulas were as follows:

6 $$\left\{ {\begin{array}{*{20}{c}} {{a_j}={a_j}+\eta {H_j}\left( {1 - {H_j}} \right)\sum\limits_{{k=1}}^{m} {{\omega _{jk}}{e_k}} } \\ {{b_k}={b_k}+{e_k}} \end{array}} \right.$$

Step 7: Determine whether the algorithm iteration had ended. This can be judged by checking if the error had reached the required precision. If not, repeat from Step 2.

**Fig. 6:**
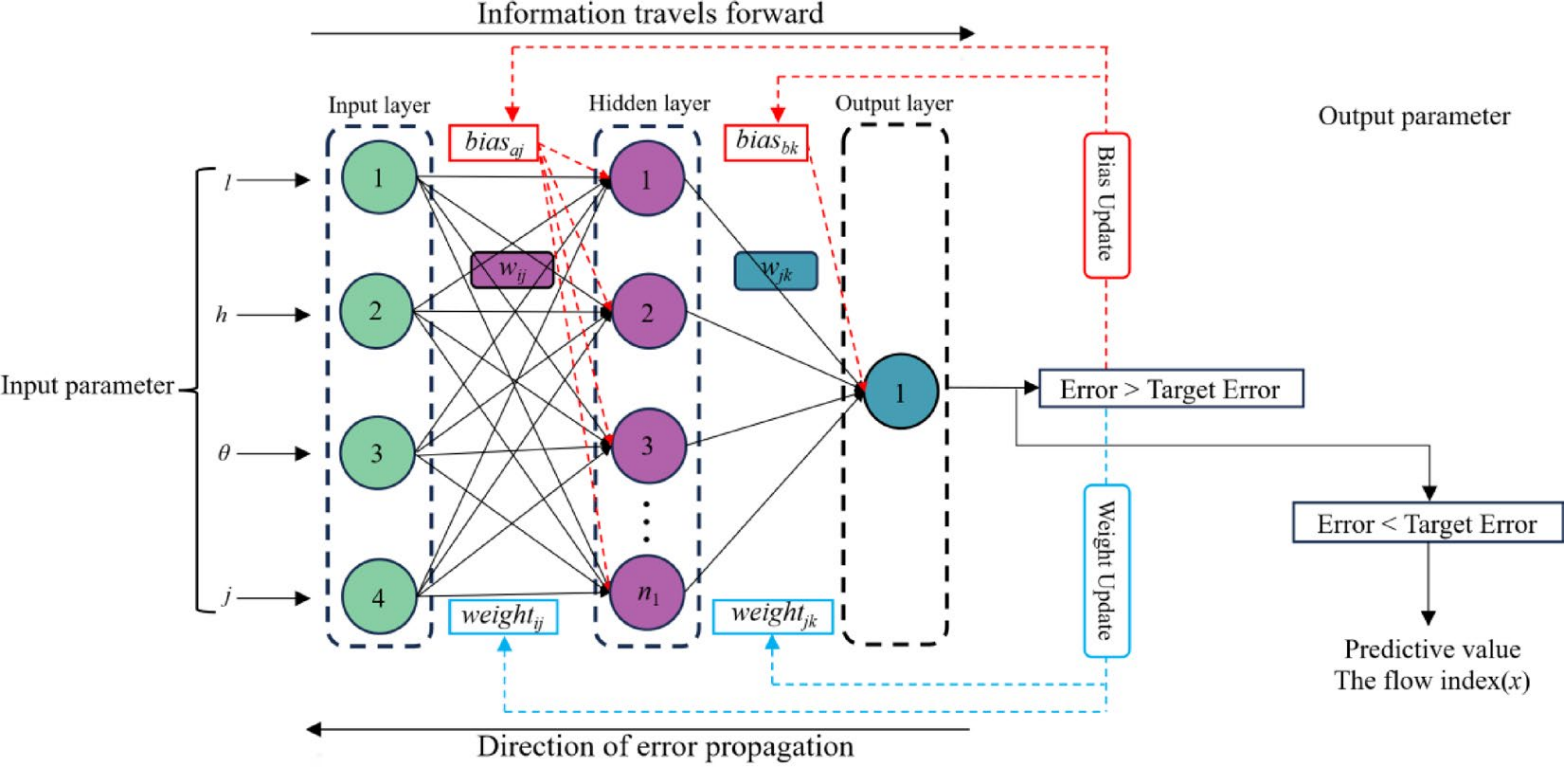
The schematic representation of the applied BP neural network model.

The structure of BP ensures efficient and accurate propagation of errors, achieving strong nonlinear mapping capability. It can prevent overfitting and underfitting, boost computational efficiency, and meet the demands of diverse application scenarios. One hidden layer and two hidden layers were set respectively for the BP, denoted as BP_1_ and BP_2_. The number of nodes corresponding to the minimum MSE in each case was selected to ensure obtaining the optimal number of nodes for the performance indicators in each case. Generally, the number of neurons in the input layer was determined by influencing factors. Each influencing factor represented a neuron in the input layer. In this study, the influencing factors were determined by four structural parameters. The number of nodes in the hidden layer was determined using empirical Eq. (7). In this BP model, the range of the number of hidden-layer neurons was (3 ~ 12). A schematic diagram of the BP_1_ model structure was shown in Fig. 6.

7 $$\left\{ \begin{gathered} {n_1}<\sqrt {(m+n)} +c \hfill \\ {n_1}={\log _2}n \hfill \\ {n_1}=\sqrt {0.43mn+0.12{m^2}+2.54n+0.77m+0.86} \hfill \\ {n_1}<n - 1 \hfill \\ \end{gathered} \right.$$

In the above four formulas, *n*_1_ was the number of hidden layer nodes, *n* was the number of input layer nodes, *m* was the number of output layer nodes, where *c* was a constant belonging to (1 ~ 10).

### Particle swarm optimization algorithm model

Particle swarm optimization (PSO) is an algorithm for adjusting the hyperparameters of an ML model. The particle swarm in PSO consists of individual particles, each of which has its own position and velocity parameters. The optimized objective function is used to determine the position parameters of an individual particle.

Assume that in a D dimensional search space, there are N particles, and each particle represents a solution. The specific calculation processes for the velocity and position of the particles are as follows:

The position of the *i* - th particle is:

8 $${X_{id}}=({x_{i1}},{x_{i2}},...,{x_{iD}})$$

The velocity of the *i* - th particle is:

9 $${V_{id}}=({v_{i1}},{v_{i2}},...,{v_{iD}})$$

The fitness value of the optimal position searched by the *i* - th particle is calculated as the average of the errors between the predicted and actual values of all samples in the test set, which is:

10 $${F_i} = \frac{1}{M}\left( {\mathop \sum \limits_{i = 1}^M ({x_i} - {{\hat x}_i})} \right)$$

Substitute the position parameters of the particle into the fitness function to obtain the fitness of the current particle. The optimal position (personal best solution) searched by the *i* - th particle is:

11 $${P_{id}}_{{,pbest}}=({p_{i1}},{p_{i2}},...,{p_{iD}})$$

The optimal position searched by the swarm (global best solution) is:

12 $${P_{d,gbest}}=({p_{1,gbest}},{p_{2,gbest}},...,{p_{D,gbest}})$$

When the two optimal values are obtained, the particle updates its velocity and position according to Eq. (13).

13 $$\left\{ {\begin{array}{*{20}{c}} {v_{{id}}^{{k+1}}=wv_{{id}}^{k}+{c_1}{r_1}\left( {P_{{id,pbest}}^{k} - x_{{id}}^{k}} \right)+{c_2}{r_2}\left( {P_{{d,gbest}}^{k} - x_{{id}}^{k}} \right)} \\ {x_{{id}}^{{k+1}}=x_{{id}}^{k}+v_{{id}}^{{k+1}}} \end{array}} \right.$$

Among them, *D* is the particle dimension; *k* is the number of iterations; *w* is the inertia weight, *c*_1_, *c*_2_ are the learning factors, *r*_1_, *r*_2_ are random numbers; $v_{{id}}^{k}$ is the velocity vector of the *i* - th particle in the *d* - th dimension at the *k* - th iteration; $x_{{id}}^{k}$ is the position vector of the *i* - th particle in the *d* - th dimension at the *k* - th iteration; *M* is the number of samples in the training set; $P_{{id,pbest}}^{k}$ is the optimal solution obtained by the *i*- th particle in the *d*- th dimension at the *k* - th iteration; $P_{{d,gbest}}^{k}$ is the optimal solution obtained by the entire particle swarm in the *d* - th dimension at the *k* - th iteration.

The influence of different hyperparameters on the performance of the neural network is significant. This study used the PSO algorithm to determine the optimal weights and thresholds of the BP. Before this process, the hyperparameters of the PSO optimization algorithm must be set. *c*_1_ and *c*_2_ were set to 2.0 and 2.0 respectively. *r*_1_ and *r*_2_ were set to 0.2 and 0.8 respectively. When initializing the PSO, the number of particles was 30, and the number of iterations for each particle was 150.

**Fig. 7:**
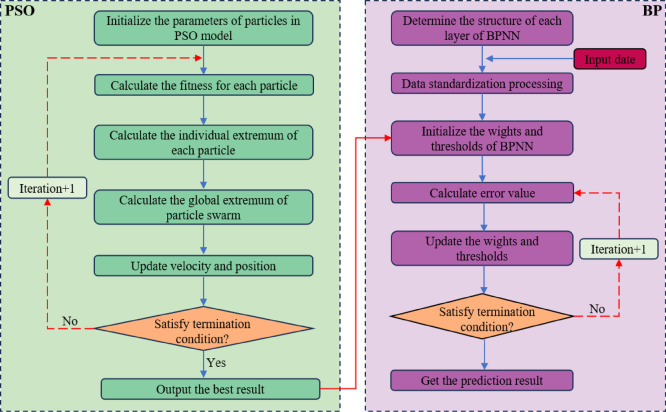
Flowchart for PSO algorithm optimization BP.

In the BP model, depending on different scenarios of the hidden layer, it was divided into two cases: BP_1_ and BP_2_. After optimization by PSO, there were a total of four combinations (BP_1_, BP_2_, BP_1_-PSO, BP_2-_PSO) used for training the ML model. The flowchart of BP-PSO was shown in Fig. 7.

### Data preprocessing and hyperparameter setting

The sample data should be preprocessed to improve the accuracy and effectiveness of the ML model before training and testing. ML is affected by the scale of input features, unifying the scale of input features is a crucial task in ML training and testing. The impact of large differences in data on prediction accuracy can be minimized by standardizing the original data. In this study, the z-score method was used to standardize the dataset. The standardization of the data was shown in Eq. (14):

14 $${x_{std}}=(x - \mu )/\sigma$$

Among them, *x*_std_ is the standardized value, *x* is the original feature value, *µ* is the mean of the data samples, and *σ* is the standard deviation of the data samples.

The “fitrnet” function was used to train the BP model, and the hyperparameters were set to their default values. “LayerSizes” was divided into two types of hidden layers: BP_1_ possessed one hidden layer, with node counts ranging from 3 to 12 for experimentation; BP_2_ possessed two hidden layers, with node counts in each hidden layer ranging from 3 to 12 for experimentation. The “ReLU” activation function was employed, with L2 regularization applied at a strength of 0.001. The iteration limit was set to 1000, and “Standardize” was configured as “true”. The programming language used for model construction was C.

### Model prediction evaluation indicator

After the construction of the ML prediction model was completed, The mean absolute error (MAE), mean squared error (MSE), root mean square error (RMSE), mean absolute percentage error (MAPE), uncertainty at 95% (U_95_), coefficient of determination (R^2^) and global performance indicator (GPI) were used to evaluate the prediction effects of BP_1_, BP_2_, BP_1_-PSO, BP_2_-PSO and other algorithms. The specific calculation methods and ranges were shown in Table 4:

**Table 4:** Mathematical formulas for evaluation indicators of machine learning prediction models.

Metrics	Equation	Description
*MAE*	$MAE=\frac{1}{n}\sum\limits_{{i=1}}^{n} {\left| {{x_i} - {{\hat {x}}_i}} \right|}$	*MAE* is an evaluation indicator used to measure the difference between predicted values and actual values. The smaller the *MAE* value, the better the performance of the prediction model, with zero being the ideal situation.
*MSE*	$MSE=\frac{1}{n}\sum\limits_{{i=1}}^{n} {{{({x_i} - {{\hat {x}}_i})}^2}}$	*MSE* is a commonly used indicator for measuring the prediction error of a model. The closer the *MSE* value is to 0, the better the performance of the prediction model.
*RMSE*	$RMSE=\sqrt {\frac{1}{n}\sum\limits_{{i=1}}^{n} {{{({x_i} - {{\hat {x}}_i})}^2}} }$	*RMSE* is the square root of the ratio of the sum of the squares of the errors between the predicted values and the actual values to the number of prediction samples. It can reflect the prediction accuracy of the model. The smaller the value of *RMSE*, the better the performance of the model.
*MAPE*	$MAPE=\frac{1}{n}\sum\limits_{{i=1}}^{n} {\left| {\frac{{{x_i} - {{\hat {x}}_i}}}{{{x_i}}}} \right|} *100\%$	*MAPE* is the percentage of the average absolute value of prediction error to the absolute value of the actual value. The lower the *MAPE* value, the better the model performance.
*U*_95_	${U_{95}}=1.96\sqrt {(SD_{{residual}}^{2}+RMS{E^2})}$	*U*_*95*_ refers to the extended uncertainty at a 95% confidence level. *U*_*95*_ represents the range of uncertainty in the measurement results, and the smaller the value of *U*_*95*_, the better the performance of the model.
*R* ^2^	${R^2} = 1 - \frac{{\sum\limits_{i = 1}^n {{{({x_i} - {{\hat x}_i})}^2}} }}{{\sum\limits_{i = 1}^n {{{({x_i} - {{\bar x}_i})}^2}} }}$	*R*^*2*^ is a measure of the model’s fit to the observed values. The range of *R*^*2*^ values is between 0 and 1. The larger *R*^*2*^, the better the regression model fits the observed values.
*GPI*	$GPI=\sqrt {{e^{\log (MAE \times RMSE \times MAPE \times {U_{95}} \times (1 - {R^2}))}}}$	*GPI* is composed of the following five indicators, mean absolute error (MAE), root mean square error (RMSE), mean absolute percentage error (MAPE), 95% confidence interval uncertainty (U_95_) and coefficient of determination (R²). These indicators are combined into a whole. The smaller the *GPI*, the better the model performance.

Note: *x*_*i*_ was the true value; ${\hat {x}_i}$ was the predicted value; ${\bar {x}_i}$ was the average value; $S{D_{residual}}$ was the standard deviation of the residuals, which represented the degree of deviation between the measured values and the predicted values.

## Results

### Statistical parameters of the training-testing datasets and determination of the hidden layer

In the training-testing dataset, the flow index of 160 test schemes ranged from 0.4545 to 0.5014 (Table 5). The flow index of the traditional labyrinth flow channel was 0.5 ~ 0.7^30^, the flow index had decreased. The parameter statistics of the data in the training-testing dataset (Tables 1 and 5) were shown in Table 6.

**Table 5:** Flow index of the training-testing dataset.

level	x	Level	x	Level	x	Level	x	Level	x
1	0.4808	33	0.4775	65	0.4900	97	0.4820	129	0.4789
2	0.4705	34	0.4748	66	0.4680	98	0.4929	130	0.4752
3	0.4874	35	0.4800	67	0.4743	99	0.4907	131	0.4812
4	0.4682	36	0.4767	68	0.4768	100	0.4819	132	0.4718
5	0.481	37	0.4616	69	0.4670	101	0.4877	133	0.4887
6	0.4676	38	0.4642	70	0.4642	102	0.4889	134	0.4718
7	0.4705	39	0.4808	71	0.4692	103	0.4618	135	0.4938
8	0.4751	40	0.4760	72	0.4699	104	0.4746	136	0.5014
9	0.4694	41	0.4669	73	0.4672	105	0.4825	137	0.4825
10	0.4742	42	0.4715	74	0.4767	106	0.4929	138	0.4919
11	0.4777	43	0.4718	75	0.4940	107	0.4767	139	0.4822
12	0.4695	44	0.4730	76	0.4901	108	0.4927	140	0.4932
13	0.4586	45	0.4680	77	0.4993	109	0.4900	141	0.4876
14	0.4833	46	0.4783	78	0.4730	110	0.4794	142	0.4953
15	0.4639	47	0.4707	79	0.4845	111	0.4884	143	0.4902
16	0.4654	48	0.4707	80	0.4772	112	0.4878	144	0.4897
17	0.4789	49	0.4967	81	0.4745	113	0.4819	145	0.4849
18	0.4654	50	0.4849	82	0.4800	114	0.4842	146	0.4784
19	0.4852	51	0.4545	83	0.4715	115	0.4726	147	0.4857
20	0.4876	52	0.4865	84	0.4870	116	0.4849	148	0.4782
21	0.4840	53	0.4745	85	0.4791	117	0.4784	149	0.4723
22	0.4798	54	0.4792	86	0.4819	118	0.4731	150	0.4865
23	0.4745	55	0.4754	87	0.4794	119	0.4769	151	0.4754
24	0.4862	56	0.4736	88	0.4790	120	0.4843	152	0.4707
25	0.4796	57	0.4653	89	0.4665	121	0.4775	153	0.4841
26	0.4753	58	0.4762	90	0.4734	122	0.4637	154	0.4735
27	0.4697	59	0.4791	91	0.4667	123	0.4761	155	0.4776
28	0.4905	60	0.4658	92	0.4762	124	0.4689	156	0.4666
29	0.4830	61	0.4651	93	0.4804	125	0.4644	157	0.4865
30	0.4783	62	0.4883	94	0.4746	126	0.4848	158	0.4726
31	0.4771	63	0.4601	95	0.4913	127	0.4896	159	0.4760
32	0.4753	64	0.4776	96	0.4846	128	0.4846	160	0.4867

The average value of the flow channel angle *θ* was 108.61, the average value of the pit depth l was 1.4, the average value of the inner and outer boundary spacing *h* was 0.6, the average value of the pit aperture *j* was 0.8, and the minimum average value of the flow index *x* was 0.4784. The maximum and minimum values of the standard deviation (SD) of the flow channel angle *θ* and the flow index *x* were 6.52 and 0.01 respectively. Among the four input parameters, the inner and outer boundary spacing *h* had the largest coefficient of variation (CV = 0.24), followed by the pit aperture *j*, the pit depth *l*, and the flow channel angle *θ*, indicating that the four input parameters had a low degree of dispersion, which improved the accuracy of the model prediction. The highest and lowest skewness coefficient (Sk) values of the four input parameters were 0.24 and − 0.05 respectively, and the skewness values of all parameters were in the range of −0.5 to 0.5, indicating that the data distribution was approximately symmetric. The kurtosis coefficients (K) of the four input parameters were between − 0.42 and − 1.39 (less than 3), and the peak of the data was flatter than that of the normal distribution, which can reduce the risk of over-fitting of the model.

**Table 6:** Statistical parameters of input and output datasets used in the prediction model (160 datasets).

Variable	Min.	Max.	Median	Mean	SD	CV	Sk.	K.
Tooth spacing, *l*(mm)	1.2	1.6	1.4	1.37	0.15	0.11	0.20	1.39
Inner and outer boundary spacing, *h*(mm)	0.4	0.8	0.6	0.58	0.14	0.24	0.24	−1.14
Flow channel angle, *θ*(°)	100	120	110	108.61	6.52	0.06	0.21	−1.08
Tooth stagger value, *j*(mm)	0.6	1.0	0.8	0.81	0.14	0.17	−0.05	−1.26
The flow index, *x*	0.4545	0.5014	0.4782	0.4784	0.01	0.02	0.02	−0.42

Note: Min., Max., Median, Mean, SD, CV, Sk., and K. represented the maximum value, minimum value, average value, median, standard deviation, coefficient of variation, skewness coefficient, and kurtosis coefficient of each variable respectively.

The optimal number of hidden layer nodes can maintain the accuracy of the model while avoiding over-fitting. In this study, the number of hidden layer nodes was gradually increased from 3 to 12, and the changes in the MSE and R^2^ of the model were observed. The MSE reaches the minimum value and the R^2^ reaches the maximum value, the optimal number of hidden nodes can be determined.

**Fig. 8:**
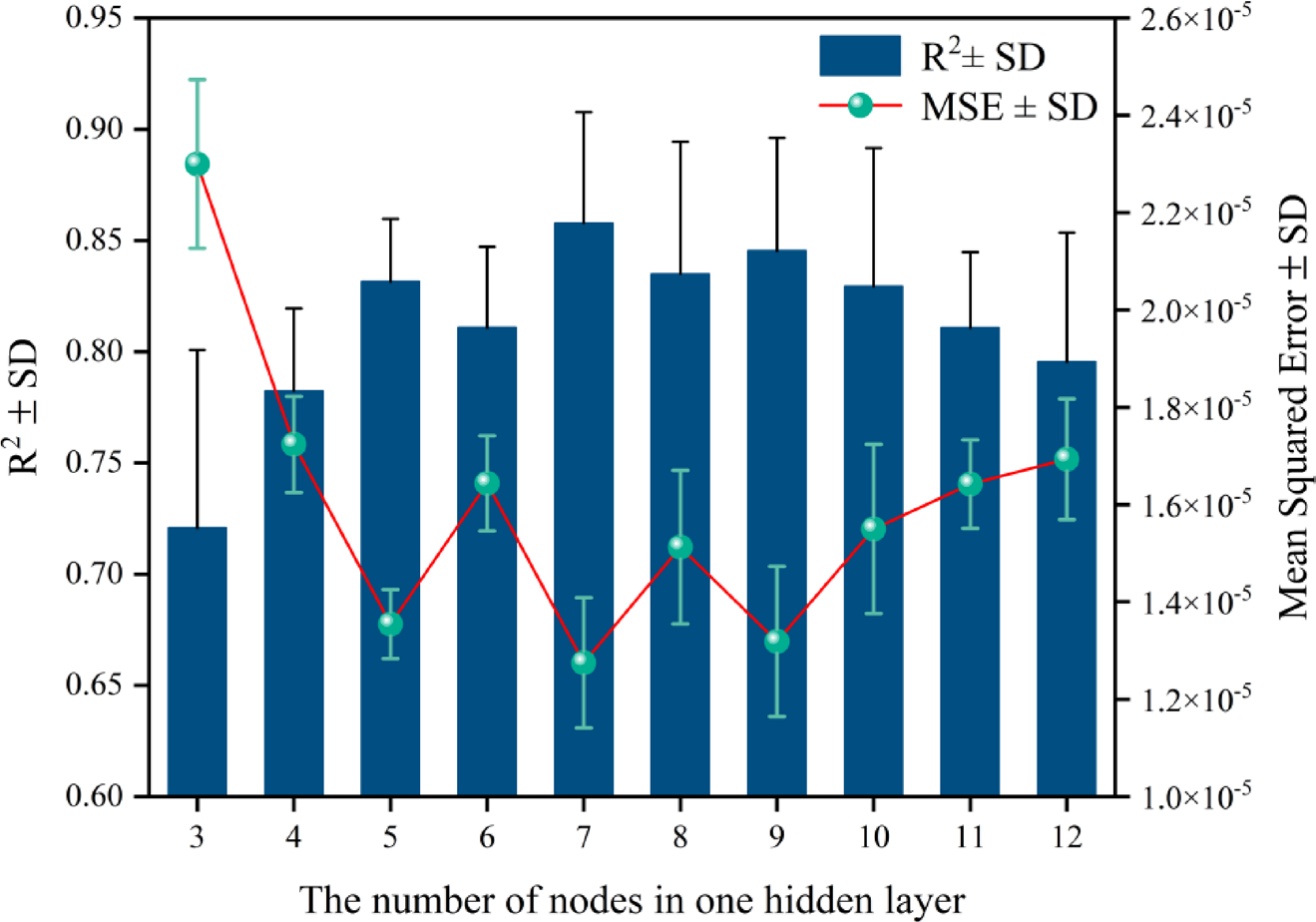
The number of nodes on training performance of BP_1_.

Figure 8 showed the relationship among the average R^2^, MSE and the number of hidden-layer nodes during 5 training sessions of the BP_1_ model. The range of R^2^ was 0.728 ~ 0.8578, and the difference between upper and lower was 0.1298. The number of hidden layers was 1, and the R² value of BP_1_ remained highly stable with minimal fluctuations. The range of MSE was 1.27 × 10^− 5^ ~ 2.30 × 10^− 5^. The number of BP_1_ nodes was 7, R²= 0.8578, and MSE = 1.27 × 10^− 5^, indicating that the model achieved optimal training performance. The optimal neural network structure of BP_1_ was set as 4–7-1.

**Fig. 9:**
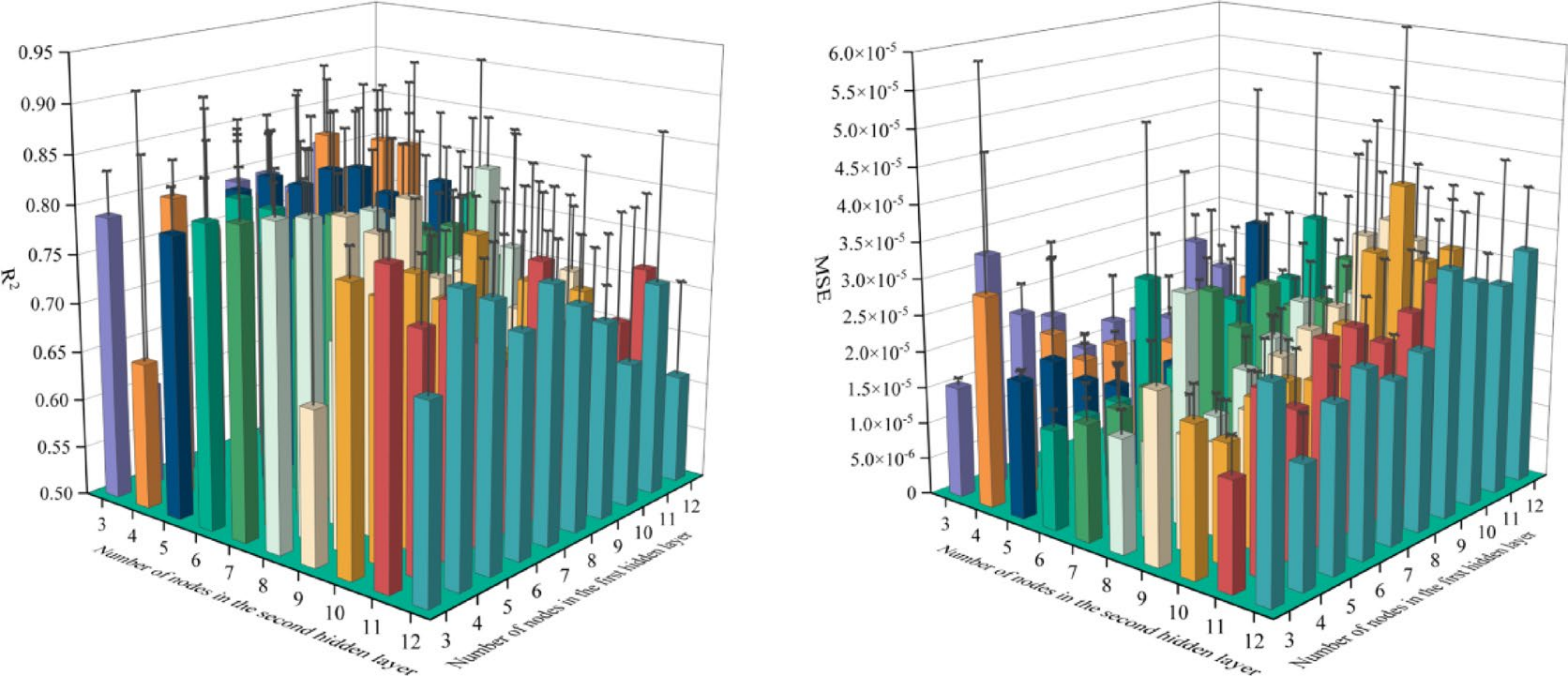
The number of nodes on training performance of BP_2_.

Figure 9 showed the relationship among the average R^2^, MSE and the number of hidden-layer nodes during 5 training sessions of the BP_2_ model. The R² value ranged from 0.6042 to 0.8344, and the difference between upper and lower was 0.2302. The number of hidden layers was 2, and the R² value of BP_2_ was unstable and fluctuates significantly. The range of MSE was 1.28 × 10⁻⁵ ~ 4.17 × 10⁻⁵. The number of hidden layer nodes in the first level was 4, the number of hidden layer nodes in the second level was 9, R² = 0.8344, and MSE = 1.28 × 10^− 5^, indicating that the model achieved optimal training performance. The optimal neural network structure of BP_2_ was set as 4-4-9-1.

### Model prediction and performance analysis

The statistical results of the performance of the four models (BP_1_, BP_2_, BP_1_-PSO, BP_2_-PSO) in predicting the flow index during the training and testing phases were shown in Table 7. Different hidden layers and model structures exhibited differences in the prediction performance of the flow index. During the training phase, the R^2^ values of the four models (BP_1_, BP_2_, BP_1_-PSO, BP_2_-PSO) for predicting the flow index were 0.9227, 0.9357, 0.9419, and 0.9533 respectively. The MSE values were 6.71 × 10^− 6^, 5.90 × 10^− 6^, 4.37 × 10^− 6^ and 3.51 × 10^− 6^ respectively. The MAE values were 1.93 × 10^− 3^, 1.80 × 10^− 3^, 1.49 × 10^− 3^ and 1.36 × 10^− 3^ respectively. The above-mentioned indicators indicated that the four models had good prediction accuracy and stability. Among them, the BP_2_-PSO model showed the best performance.

**Table 7:** Training and testing performance of four models for the flow index of pit drip irrigation emitters.

Running Tasks	Metrics	BP_1_	BP_2_	BP_1_-PSO	BP_2_-PSO
Training	R^2^	0.9227	0.9357	0.9419	0.9533
	MSE	6.71 × 10^− 6^	5.90 × 10^− 6^	4.37 × 10^− 6^	3.51 × 10^− 6^
	MAE	1.93 × 10^− 3^	1.80 × 10^− 3^	1.49 × 10^− 3^	1.36 × 10^− 3^
	MAPE	3.69 × 10^− 3^	3.05 × 10^− 3^	2.92 × 10^− 3^	2.66 × 10^− 3^
	RMSE	2.59 × 10^− 3^	2.42 × 10^− 3^	2.09 × 10^− 3^	1.87 × 10^− 3^
	U_95_	5.11 × 10^− 3^	4.76 × 10^− 3^	4.24 × 10^− 3^	3.68 × 10^− 3^
	GPI	3.82 × 10^− 3^	3.36 × 10^− 3^	2.95 × 10^− 3^	2.56 × 10^− 3^
Testing	R^2^	0.8379	0.8687	0.8931	0.9456
	MSE	1.21 × 10^− 5^	1.15 × 10^− 5^	7.97 × 10^− 6^	5.49 × 10^− 6^
	MAE	2.70 × 10^− 3^	2.56 × 10^− 3^	2.14 × 10^− 3^	1.98 × 10^− 3^
	MAPE	5.70 × 10^− 3^	5.31 × 10^− 3^	4.55 × 10^− 3^	4.09 × 10^− 3^
	RMSE	3.48 × 10^− 3^	3.39 × 10^− 3^	2.82 × 10^− 3^	2.34 × 10^− 3^
	U_95_	8.75 × 10^− 3^	9.36 × 10^− 3^	7.68 × 10^− 3^	6.46 × 10^− 3^
	GPI	6.35 × 10^− 3^	5.96 × 10^− 3^	4.88 × 10^− 3^	3.74 × 10^− 3^

After training four models, the remaining 15% of the dataset was used to test the accuracy of the models. During the testing phase, the R^2^ values for predicting the flow index of the four models (BP_1_, BP_2_, BP_1_-PSO, BP_2_-PSO) were 0.8379, 0.8687, 0.8931 and 0.9456 respectively. The MSE were 1.21 × 10^− 5^, 1.15 × 10^− 5^, 7.97 × 10^− 6^ and 5.49 × 10^− 6^ respectively. The MAE were 2.70 × 10^− 3^, 2.56 × 10^− 3^, 2.14 × 10^− 3^ and 1.98 × 10^− 3^ respectively. The MAPE were 5.70 × 10^− 3^, 5.31 × 10^− 3^, 4.55 × 10^− 3^ and 4.09 × 10^− 3^ respectively. The RMSE were 3.48 × 10^− 3^, 3.39 × 10^− 3^, 2.8210^−3^ and 2.34 × 10^− 3^ respectively. Among them, BP_2_-PSO showed the best performance among the four models in the test set.

The PSO optimization algorithm played an important role in improving the accuracy of BP model in predicting the flow index of pit drip irrigation emitters. The comparison results of four models showed that all models can accurately predict the flow index of pit drip irrigation emitters. The R^2^ values of the four models tested were all above 0.8. The R^2^ predicted by the BP-PSO model was greater than that of the BP model.

**Fig. 10:**
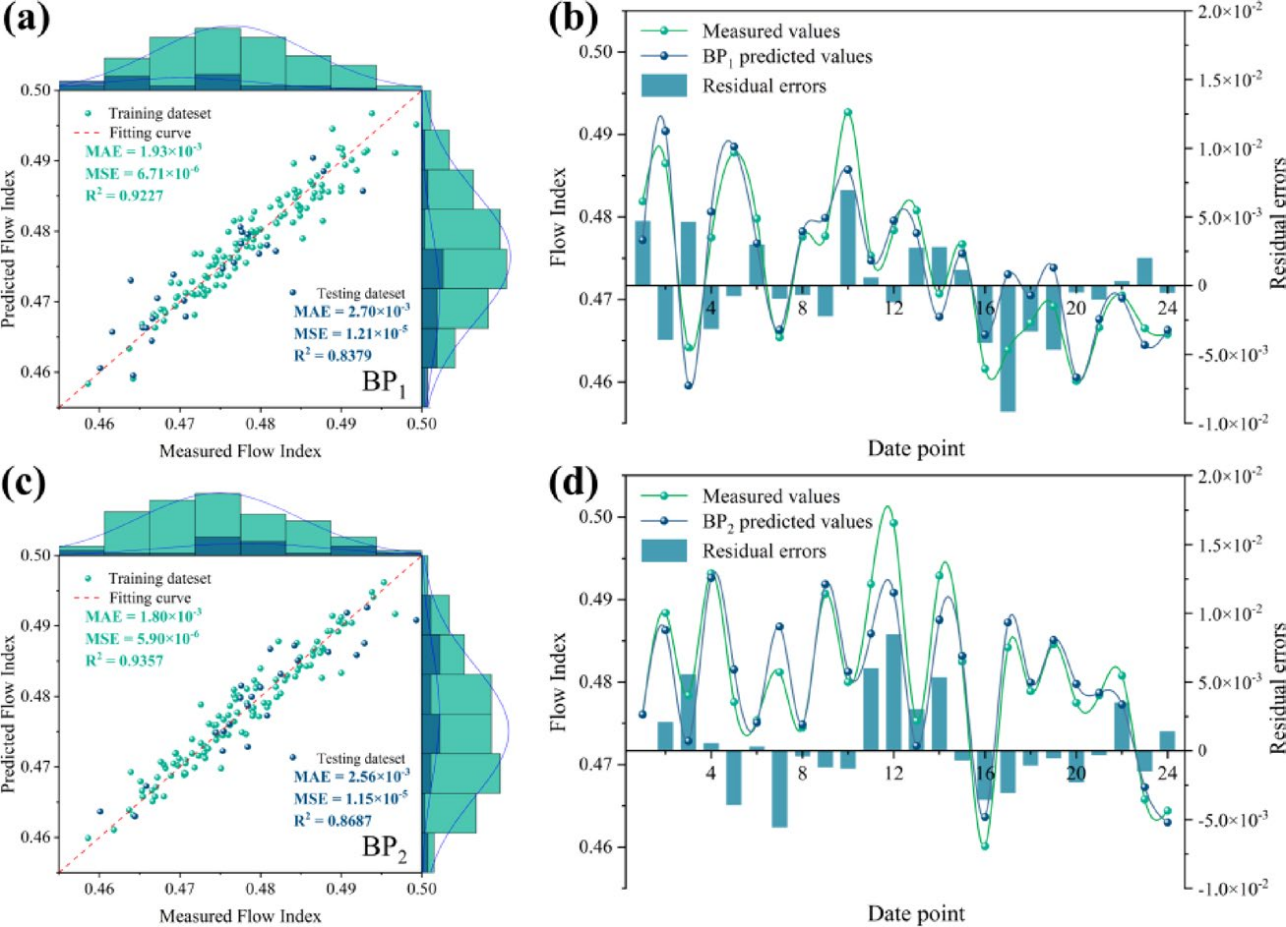
The marginal plot and prediction results of the flow index for BP experimental and predicted values.

Figure 10 (a) and (c) showed the edge histograms on the training-testing dataset, where the scatter plot and histogram described the scatter distribution and frequency distribution of the predicted and measured flow index. The predictive performance of the model on output parameters can be visually compared between the two figures. The flow index distribution range of the BP_1_ model training set was 0.4545 ~ 0.5014, and its predicted flow index range was 0.4567 ~ 0.4994. The flow index distribution range of the BP_2_ model training dataset was 0.4545 ~ 0.5014, and its predicted flow index range was 0.4599 ~ 0.4979. From the scatter plot, it can be seen that the flow index error data of the BP_1_ model had a wider range of discreteness than that of the BP_2_ model. The frequency of training data of BP_1_ being close to the 45-degree diagonal was higher than that of test data, and the measured values and predicted values were basically distributed between 0.4600 and 0.4950. The frequency of training data of BP_2_ near the 45-degree diagonal was higher than that of test data, and the measured values and predicted values were basically distributed between 0.4600 and 0.4950. The flow index values predicted by the BP_2_ model were more closely clustered around the diagonal than those predicted by the BP_1_ model, indicating that the prediction accuracy of the BP_2_ model was higher.

Figure 10 (b) and (d) showed the measured flow index data and BP_1_ and BP_2_ model prediction data on the training-testing dataset, as well as their error values. In the two figures, it can be observed that the measured values in the BP_2_ model had a high degree of fit with the predicted flow index scatter points, and the residual values were relatively small. The flow index distribution range of the measured values of the BP_1_ model was 0.4601 ~ 0.4927, the flow index distribution range of the predicted values was 0.4596 ~ 0.4904, and the residual flow index distribution range was − 9.15 × 10^− 3^ ~ 6.96 × 10^− 3^. The flow index distribution range of the measured values of the BP_2_ model was 0.4601 ~ 0.4993, the flow index distribution range of the predicted values was 0.4630 ~ 0.4926, and the residual flow index distribution range was − 5.54 × 10^− 3^ ~ 8.45 × 10^− 3^.

**Fig. 11:**
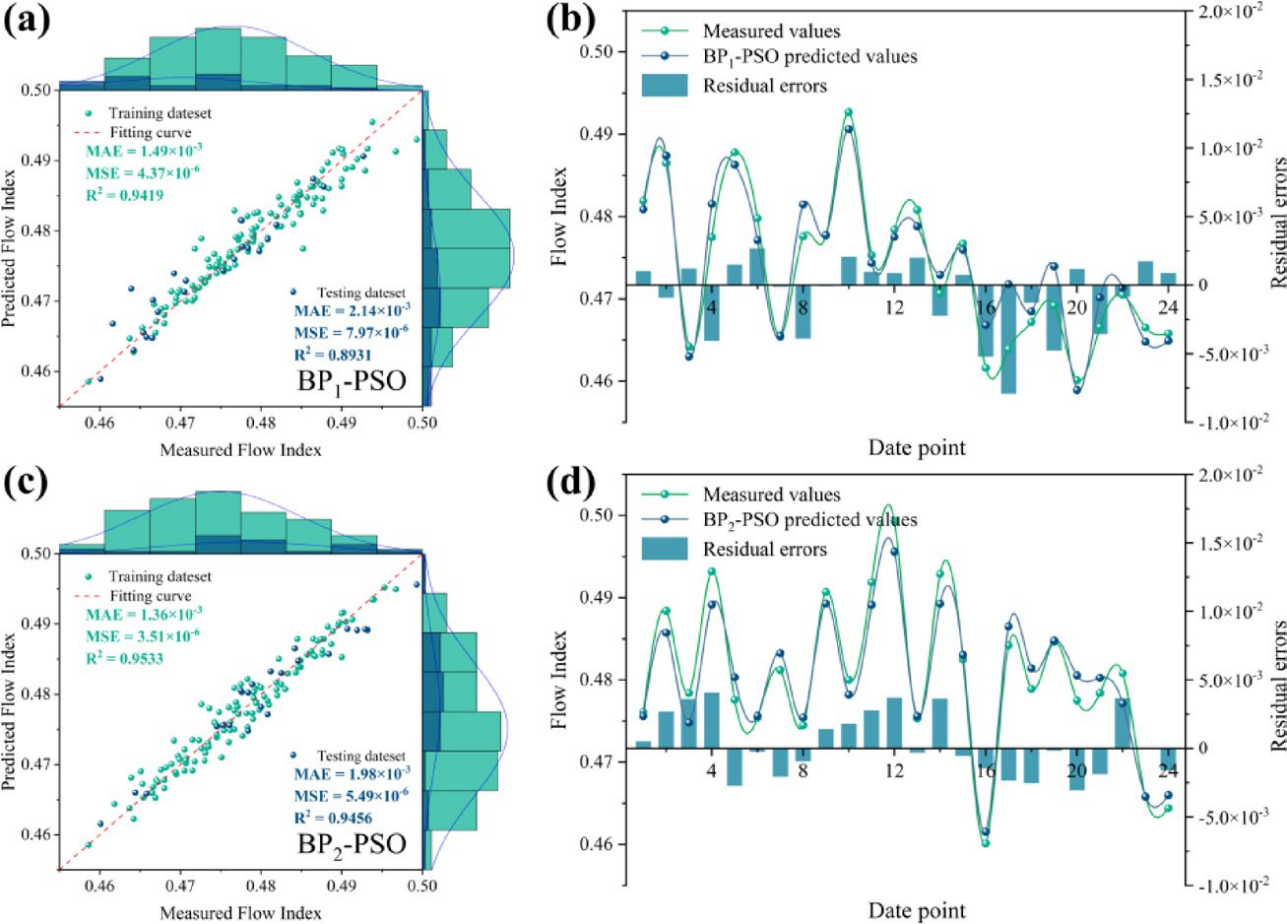
The marginal plot and prediction results of the flow index for BP-PSO experimental and predicted values.

Figure 11 (a) and (c) showed the edge histograms on the training-testing dataset, where the scatter plot and histogram depicted the scatter distribution and frequency distribution of the predicted and measured flow index. The flow index distribution range of the BP_1_-PSO model training dataset was 0.4545 ~ 0.5014, and its predicted flow index range was 0.4559 ~ 0.4997. The flow index distribution range of the BP_2_-PSO model training dataset was 0.4545 ~ 0.5014, and its predicted flow index range was 0.4585 ~ 0.5010. From the scatter plot, it can be seen that the flow index error data of the BP_1_-PSO model had a wider range of discreteness than that of the BP_2_-PSO model. The frequency of training data of BP_1_-PSO being close to the 45-degree diagonal was higher than that of test data, and the measured values and predicted values were basically distributed between 0.4650 and 0.4900. The frequency of training data of BP_2_-PSO near the 45-degree diagonal was higher than that of test data, and the measured values and predicted values were basically distributed between 0.4600 and 0.4950. The flow index values predicted by the BP_2_-PSO model are were closely clustered around the diagonal than those predicted by the BP_1_-PSO model, indicating that the prediction accuracy of the BP_2_-PSO model was higher.

Figure 11 (b) and (d) showed the measured data of the flow index, the predicted data of the BP_1_-PSO and BP_2_-PSO models, and the magnitude of their error values on the training-testing dataset. In the two figures, it can be observed that the measured values and predicted values of the flow index in the BP_2_-PSO model had a high degree of scatter fit, and the residual values were relatively small. The flow index distribution range of the measured values of the BP_1_-PSO model was 0.4601 ~ 0.4927, the flow index distribution range of the predicted values was 0.4589 ~ 0.4906, and the residual flow index distribution range was − 7.89 × 10^− 3^~2.66 × 10^− 3^. The flow index distribution range of the measured values of the BP_2_-PSO model was 0.4601 ~ 0.4993, the flow index distribution range of the predicted values was 0.4515 ~ 0.4956, and the residual flow index distribution range was − 3.04 × 10^− 3^~4.06 × 10^− 3^.

**Fig. 12:**
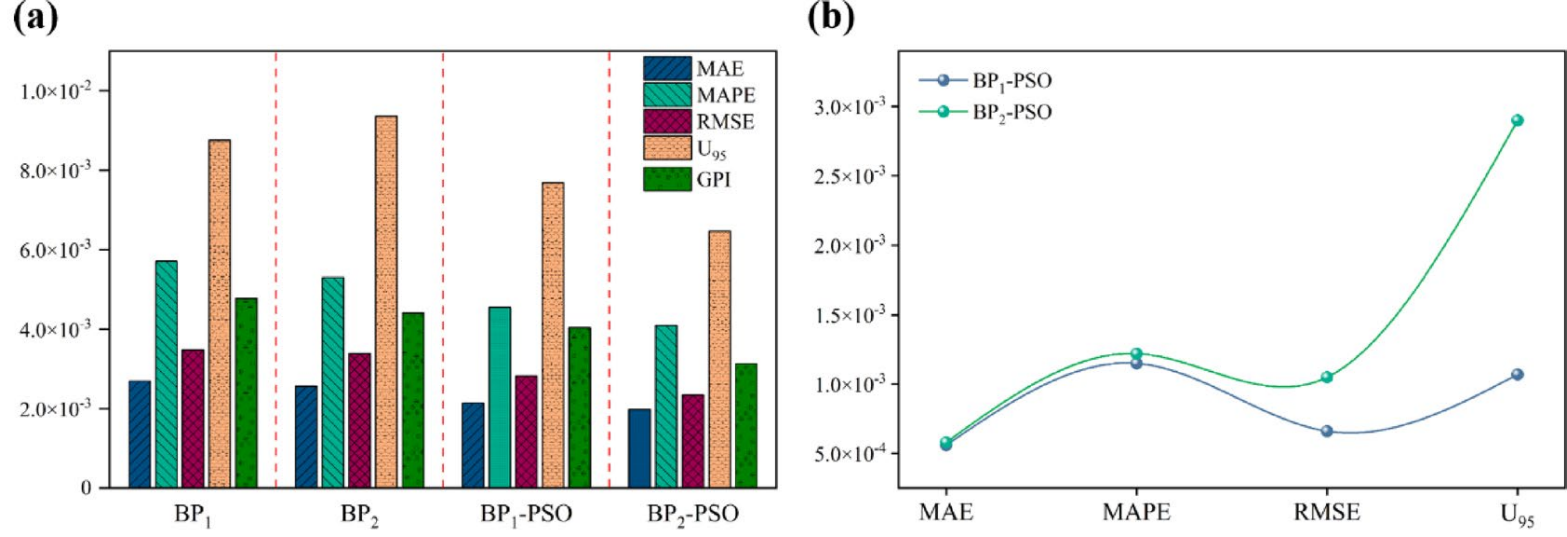
**(a)** Main predictive performance bar charts of BP and BP-PSO. **(b)** BP-PSO prediction error indicators and their reduction amounts.

Figure 12 (a) and (b) showed a comparison of the prediction error indicator and their reductions for BP_1_ and BP_2_ models. Compared with the results of the BP_1_ model, the MSE and MAE performance of BP_1_-PSO improved by 34.87% and 22.80% during the training phase, and the MAE, MAPE, and RMSE performance of BP_1_-PSO improved by 20.74%, 20.17%, and 18.97% during the testing phase; Compared with the results of the BP_2_ model, the MSE and MAE performance of BP_2_-PSO improved by 40.51% and 22.66% during the training phase, and the MAE, MAPE, and RMSE performance of BP_2_-PSO improved by 22.66%, 22.98%, and 30.97% during the testing phase. The above results verified the feasibility and applicability of PSO algorithm in optimizing BP model. The BP-PSO model had higher prediction accuracy than the BP model, and the BP_2_-PSO model had the best prediction performance among the four ML models.

This study introduced U_95_ and GPI for comprehensive evaluation. The U_95_ of BP_1_, BP_2_, BP_1_-PSO and BP_2_-PSO were 8.75 × 10^− 3^, 9.36 × 10^− 3^, 7.68 × 10^− 3^, and 6.46 × 10^− 3^ respectively. The uncertainty of all four models was less than 0.01, indicating good prediction accuracy. The GPI of BP_1_, BP_2,_ BP_1_-PSO and BP_2_-PSO were 6.35 × 10^− 3^, 5.96 × 10^− 3^, 4.88 × 10^− 3^, and 3.74 × 10^− 3^ respectively. The GPI of the four models were close to 0, indicating good prediction accuracy. The above results indicated that the prediction accuracy rankings of the four models were as follows: BP_2_-PSO > BP_1_-PSO > BP_2_ > BP_1_.

**Fig. 13:**
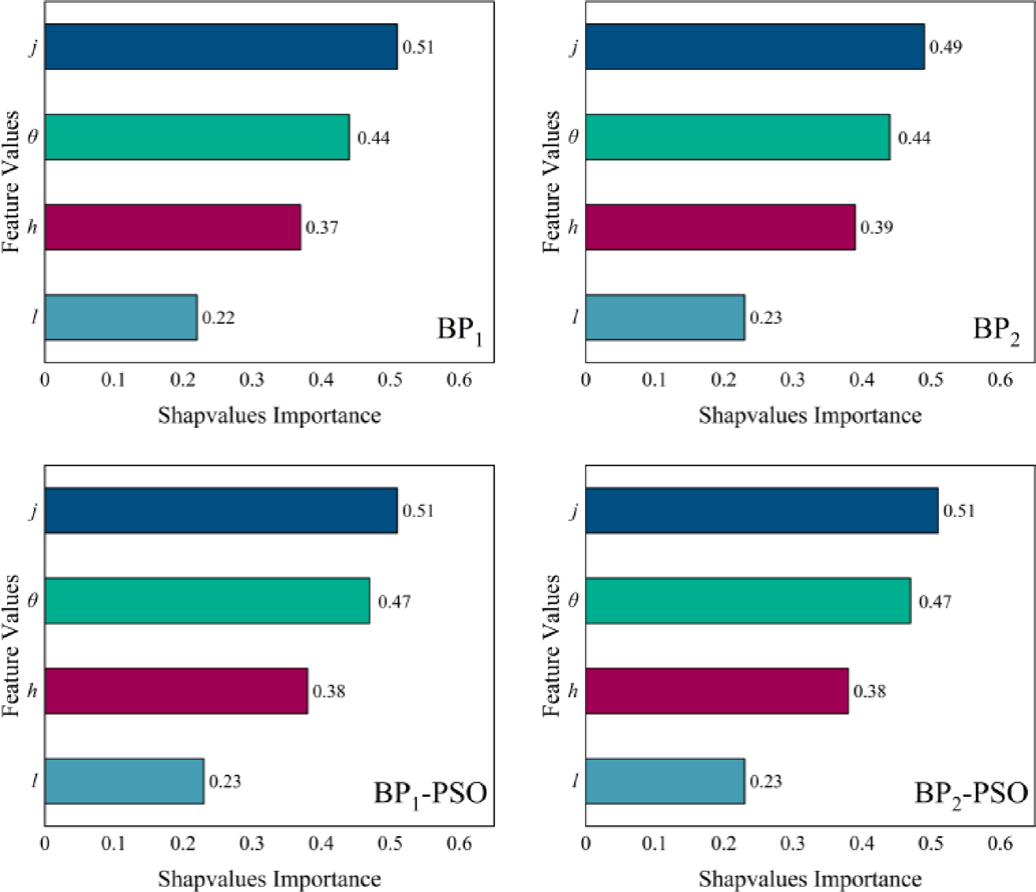
Ranking of importance of structural parameters of BP model and BP-PSO model to flow index.

SHAP analysis was used to quantitatively evaluate the influence of input parameters. BP_1_, BP_2_, BP_1_-PSO and BP_2_-PSO models were analyzed respectively, and it was found that *j* eigenvalue was better than the other three eigenvalues in importance ranking. *j* was the most critical parameter affecting the change of irrigation performance of drip irrigation emitters. The importance ranking of eigenvalues was *j* > *θ* > *h* > *l*, as shown in Fig. 13.

### External validation of the prediction model

In the previous section, internal validation was applied to evaluate the performance of four models. This section conducted external validation of four models to enhance the reliability and practicality. External validation used an external validation dataset, and Table 8 showed the predicted and experimental values of flow index for four models in the external validation dataset. The flow index distribution range of the measured values in the external validation dataset was 0.4618 ~ 0.507, the flow index distribution range of the predicted values in the BP_1_ model was 0.4638 ~ 0.5038, the flow index distribution range of the predicted values in the BP_2_ model was 0.4611 ~ 0.5024, the flow index distribution range of the predicted values in the BP_1_-PSO model was 0.4636 ~ 0.4984, and the flow index distribution range of the predicted values in the BP_2_-PSO model was 0.4644 ~ 0.5019.

**Table 8:** Predicted results and experimental values of four ML models on external validation datasets.

Level	Simulation				
	Measured	BP_1_	BP_2_	BP_1_-PSO	BP_2_-PSO
1	0.4876	0.4881	0.4854	0.4870	0.4889
2	0.4648	0.4697	0.4651	0.4682	0.4679
3	0.4858	0.4918	0.4852	0.4920	0.4832
4	0.4702	0.4731	0.4737	0.4733	0.4702
5	0.4864	0.4879	0.4754	0.4836	0.4810
6	0.4885	0.4957	0.4888	0.4905	0.4914
7	0.4767	0.4794	0.4802	0.4790	0.4777
8	0.4639	0.4679	0.4687	0.4713	0.4689
9	0.473	0.4709	0.4730	0.4726	0.4721
10	0.4818	0.4805	0.4810	0.4816	0.4818
11	0.4873	0.4872	0.4876	0.4883	0.4880
12	0.507	0.5038	0.5024	0.4984	0.5019
13	0.4694	0.4717	0.4688	0.4689	0.4696
14	0.4773	0.4726	0.4746	0.4718	0.4718
15	0.4618	0.4638	0.4611	0.4636	0.4644
16	0.4813	0.4788	0.4794	0.4799	0.4780
17	0.4878	0.4906	0.4896	0.4864	0.4872
18	0.48	0.4798	0.4824	0.4827	0.4822
19	0.4718	0.4681	0.4699	0.4705	0.4704
20	0.4811	0.4829	0.4803	0.4804	0.4802
21	0.4925	0.4855	0.4886	0.4880	0.4871
22	0.4883	0.4899	0.4901	0.4901	0.4913
23	0.4884	0.4911	0.4881	0.4881	0.4879
24	0.4736	0.4765	0.4735	0.4784	0.4743
25	0.4718	0.4819	0.4762	0.4805	0.4791

**Fig. 14:**
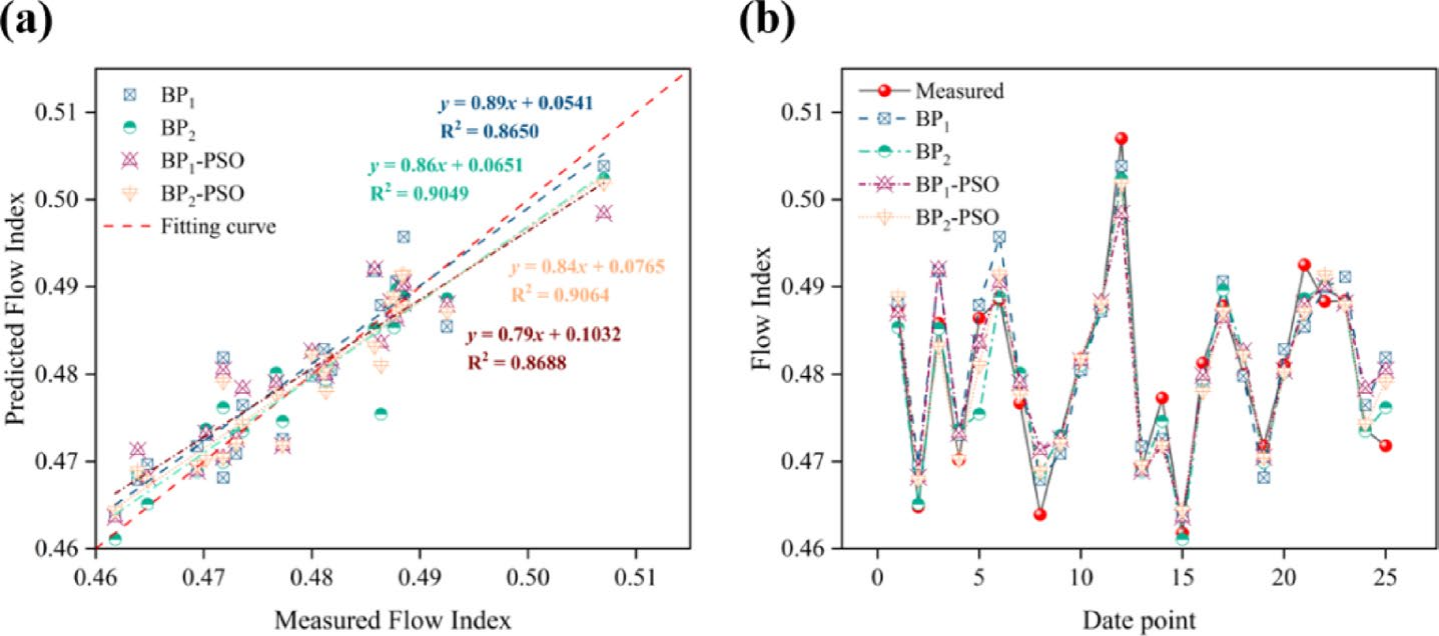
Scatter plot of external validation dataset for BP, BP-PSO model prediction and experimental and predicted values.

Figure 14 (a) and (b) showed scatter plots of experimental and predicted flow index for BP_1_, BP_2_, BP_1_-PSO and BP_2_-PSO models on an external validation dataset. In Fig. 14 (a), linear fitting (*y=kx+b*) was used to describe the relationship between the predicted results and experimental results, visually comparing the predictive performance of the four models on the output parameter (flow index). The closer the model data points were to the diagonal, the higher the prediction accuracy of the model. The slopes of BP_1_, BP_2_, BP_1_-PSO and BP_2_-PSO were 0.89, 0.86, 0.79, and 0.84 respectively. Compared with internal validation, the R^2^ values of BP_1_, BP_2_, BP_1_-PSO and BP_2_-PSO models were 0.8650, 0.9049, 0.8688, and 0.9064 respectively. The R^2^ values of all four models were greater than 0.85, indicating good generalization ability. In Fig. 14 (b), it can be observed that the measured values and predicted values in the BP_1_, BP_2_, BP_1_-PSO and BP_2_-PSO models had high scatter fit, indicating that the four models had high prediction accuracy.

**Fig. 15:**
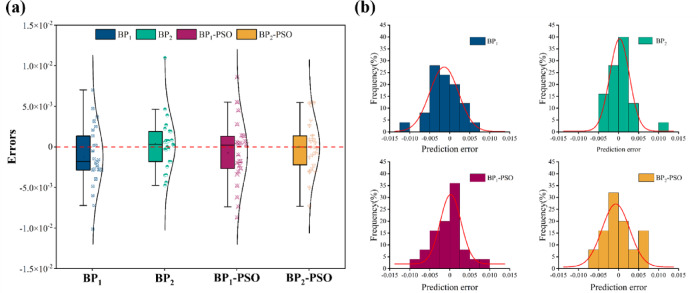
**(a)** Error distribution boxplot and **(b)** prediction error frequency distribution for the external dataset in the BP and BP-PSO models.

Figure 15 (a) showed the error distribution of flow index prediction for four models. The distribution range of the error in predicting the flow index of BP_1_ model was − 1.01 × 10^− 2^~7.00 × 10^− 3^, the distribution range of the error in predicting the flow index of BP_2_ model was − 4.80 × 10^− 3^~1.10 × 10^− 2^, the distribution range of the error in predicting the flow index of BP_1_-PSO model was − 8.70 × 10^− 3^~8.60 × 10^− 3^, and the distribution range of the error in predicting the flow index of BP_2_-PSO model was − 7.30 × 10^− 3^~5.50 × 10^− 3^. The errors of the four models were concentrated near the zero point, with a concentrated error distribution and small fluctuations. The four models had higher prediction accuracy and stability when processing different data. Figure 15 (b) showed the frequency distribution of predicted flow index error fitted to a normal distribution. The prediction error frequencies of the four models followed a normal distribution, with the prediction error of BP_1_ model concentrated between − 0.0075 and 0.0075, the prediction error of BP_2_ model concentrated between − 0.005 and 0.005, the prediction error of BP_1_-PSO model concentrated between − 0.01 and 0.01, and the prediction error of BP_2_-PSO model concentrated between − 0.0075 and 0.0075. The distribution of prediction errors for all four models was relatively concentrated, and had shown good prediction accuracy and stability in external validation of prediction models, which can adapt to the prediction needs of different datasets.

### Comparison of multiple models in ML algorithms

In order to evaluate the prediction accuracy of four models in the flow index of pit drip irrigation emitters, this study selected six classic machine learning models Categorical boosting (CatCoost), Least squares boosting (LSBoost), Multilayer perceptron support vector machine (MLP-SVM), Multilayer perceptron determination tree (MLP-DT), Gaussian regression (GR), and Multiple linear regression (MLR)) for analysis and comparison. Trained six models separately using training-testing datasets and external validation datasets, and evaluated the accuracy of model predictions using R^2^, MAE, MAPE, RMSE, U_95_ and GPI. Table 9 was showed the performance of six models in predicting the flow index of pit drip irrigation emitters. During the testing phase, six ML models (CatCoost, LSBoost, MLP-SVM, MLP-DT, GR, MLR) predicted flow index with R^2^ values of 0.8488, 0.8017, 0.8560, 0.8589, 0.8728 and 0.6103, U_95_ values of 1.08 × 10^− 2^, 1.11 × 10^− 2^, 9.55 × 10^− 3^, 9.47 × 10^− 3^, 8.21 × 10^− 3^ and 1.58 × 10^− 2^, and GPI values of 6.64 × 10^− 3^, 7.59 × 10^− 3^, 6.28 × 10^− 3^, 6.21 × 10^− 3^, 5.37 × 10^− 3^ and 1.21 × 10^− 2^ respectively. During the external validation phase, six ML models (CatCoost, LSBoost, MLP-SVM, MLP-DT, GR, MLR) predicted flow index with R^2^ values of 0.8025, 0.7302, 0.8482, 0.8928, 0.9062 and 0.4018, U_95_ values of 1.31 × 10^− 2^, 1.44 × 10^− 2^, 1.23 × 10^− 2^, 9.16 × 10^− 3^, 7.25 × 10^− 3^ and 2.21 × 10^− 2^, and GPI values of 8.61 × 10^− 3^, 9.59 × 10^− 3^, 7.73 × 10^− 3^, 5.57 × 10^− 3^, 5.22 × 10^− 3^ and 1.84 × 10^− 2^ respectively.

**Table 9:** Performance of multi-model ML algorithms in predicting the flow index of pit drip irrigation emitters.

Running Tasks	Metrics	CatCoost	LSBoost	MLP-SVM	MLP-DT	GR	MLR
Training-testing dataset	R^2^	0.8488	0.8017	0.8560	0.8589	0.8728	0.6103
	MAE	2.72 × 10^− 3^	3.04 × 10^− 3^	2.69 × 10^− 3^	2.68 × 10^− 3^	2.29 × 10^− 3^	4.47 × 10^− 3^
	MAPE	5.82 × 10^− 3^	6.38 × 10^− 3^	5.63 × 10^− 3^	5.59 × 10^− 3^	4.98 × 10^− 3^	9.35 × 10^− 3^
	RMSE	3.62 × 10^− 3^	4.06 × 10^− 3^	3.46 × 10^− 3^	3.42 × 10^− 3^	2.94 × 10^− 3^	5.69 × 10^− 3^
	U_95_	1.08 × 10^− 2^	1.11 × 10^− 2^	9.55 × 10^− 3^	9.47 × 10^− 3^	8.21 × 10^− 3^	1.58 × 10^− 2^
	GPI	6.64 × 10^− 3^	7.59 × 10^− 3^	6.28 × 10^− 3^	6.21 × 10^− 3^	5.37 × 10^− 3^	1.21 × 10^− 2^
External validation dataset	R^2^	0.8025	0.7302	0.8482	0.8928	0.9062	0.4018
	MAE	3.50 × 10^− 3^	3.57 × 10^− 3^	3.40 × 10^− 3^	2.54 × 10^− 3^	2.64 × 10^− 3^	6.99 × 10^− 3^
	MAPE	6.99 × 10^− 3^	7.12 × 10^− 3^	6.78 × 10^− 3^	5.09 × 10^− 3^	5.27 × 10^− 3^	1.39 × 10^− 2^
	RMSE	4.88 × 10^− 3^	5.14 × 10^− 3^	4.38 × 10^− 3^	3.28 × 10^− 3^	3.27 × 10^− 3^	7.93 × 10^− 3^
	U_95_	1.31 × 10^− 2^	1.44 × 10^− 2^	1.23 × 10^− 2^	9.16 × 10^− 3^	7.25 × 10^− 3^	2.21 × 10^− 2^
	GPI	8.61 × 10^− 3^	9.59 × 10^− 3^	7.73 × 10^− 3^	5.57 × 10^− 3^	5.22 × 10^− 3^	1.84 × 10^− 2^

**Fig. 16:**
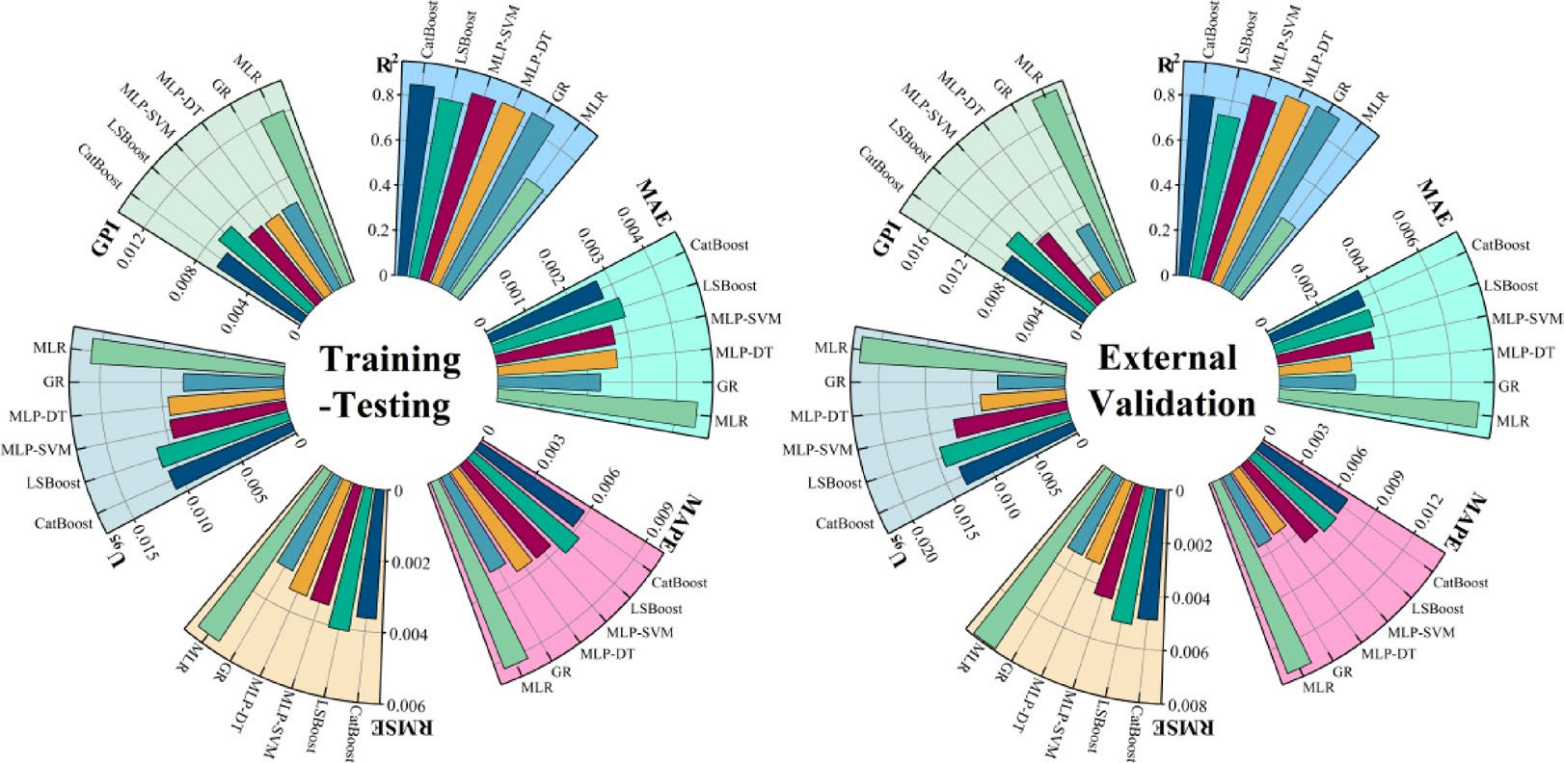
performance comparison of ML multi model algorithm in training-testing and external validation.

In external validation, the R^2^ values of CatCoost, GR, MLP-DT, and MLP-SVM models were all greater than 0.8, demonstrating excellent generalization ability. The R^2^ of the MLR model was less than 0.5, indicating that the generalization ability of model was average. The GR model among the six models had the best accuracy in predicting the flow index (Fig. 16). Compared with the four models BP_1_, BP_2_, BP_1_-PSO, and BP_2_-PSO, the prediction accuracy ranking of all models during the training-testing process was as follows: BP_2_-PSO > BP_1_-PSO > BP_2_ > GR > MLP-DT > MLP-SVM > CatBoost > BP_1_ > LSBoost > MLR. The ten models ranked as follows in terms of prediction accuracy during external validation: BP_2_ > GR > BP_2_ -PSO > MLP-DT > BP_1_-PSO > BP_1_ > MLP-SVM > CatCoost > LSBoost > MLR.

## Discussion

The current research results indicated that machine learning can become an effective tool for predicting the flow index of drip irrigation emitters based on structural parameters, which can quickly and accurately obtain the specific value of the flow index of pit drip irrigation emitters. This is a great significance for optimizing the design of drip irrigation systems, improving irrigation uniformity and anti-clogging performance^19,31^. The BP-PSO model is superior to the BP model in predicting the flow index accuracy of the pit drip irrigation emitters. PSO uses swarm intelligence parallel search to find the globally optimal combination of initial weights and thresholds for the BP network. It can effectively avoid the training traps of the traditional gradient descent method and improve the accuracy of the model, which is similar to the research results of Xiao^32^. In the BP-PSO hybrid model, PSO predict the flow index indirectly, and solves the defects of BP training such as sensitivity to initial weight and easy to trap local optimization, thus indirectly and decisively improving the accuracy and reliability of the final flow index prediction model. The R^2^ values of both BP-PSO and BP models for predicting flow index are greater than 0.9, indicating high prediction accuracy of the models^33^.

The hidden layer is the intermediate layer between the input layer and the output layer. It can integrate the input data. Through the weighted summation among neurons and operations of activation functions, the information transmitted from the input layer can be utilized by the output layer in a more appropriate way. This improves the accuracy and generalization ability of the model^34^. In this study, the optimal number of single hidden nodes and double hidden nodes were determined respectively under the k-fold cross-validation method. It was found that the prediction accuracy of the optimal double-hidden-node models (BP_2_, BP_2_-PSO) was higher than that of the single-hidden-node models (BP_1_, BP_1_-PSO). This indicated that the double hidden layer can better fit complex functional relationships, extract better features and fitting trends. It can transform the input data through more complex operations, getting closer to the true functional mapping, thus improving the accuracy of prediction or modeling^35^.

In this study, the parameters (MAE, MSE, RMSE, MAPE, U_95_, GPI) were used to evaluate the prediction effects of the flow index of four models: BP_1_, BP_2_, BP_1_-PSO and BP_2_-PSO. Among them, U_95_ can more accurately predict the actual range of the flow index of pit drip irrigation emitters by quantifying uncertain factors. The magnitude of U_95_ for the four models was 10⁻³, and the interval value was between 0.45 and 0.50, indicating high prediction accuracy and a reliable prediction range^36,37^. GPI provided a comprehensive measurement standard. It is not limited to local performance characteristics and can make a rational evaluation of model prediction. The magnitude of GPI for the four models is 10⁻³, and the smaller the value had the better the model performance.

There are differences in the prediction accuracy of different machine learning algorithms. BP-PSO and BP models had good performance in predicting the flow index of pit drip irrigation emitters. At the same time, it is necessary to compare the prediction accuracy of some classical machine learning methods to ultimately determine the overall performance of BP-PSO and BP models^38^. This study selected six models (CatBoost, LSBoost, MLP-SVM, MLP-DT, GR and MLR) for analysis and comparison. The comprehensive evaluation of the models was conducted using training-testing datasets and external validation datasets. BP_2_-PSO and BP_1_-PSO models had the highest prediction accuracy among the ten machine learning methods, BP_2_ model ranked high in prediction accuracy, and BP_1_ model ranked middle in prediction accuracy. This indicated that BP-PSO and BP models were feasible for predicting the flow index of pit drip irrigation emitters, and particle swarm optimization of neural network models can improve model accuracy.

This experiment only establishes a machine learning prediction model between the structural parameters and flow index of drip irrigation emitters and the optimization of core hydraulic performance indicators. However, the performance of emitters not only includes the flow index, but also its anti-clogging performance. This research has not yet incorporated anti-clogging performance into the modeling and optimization framework, which is an important limitation of this paper. The scope of the research at this stage is clearly limited to accurate prediction and optimization of hydraulic performance.

## Conclusion

This article established a flow index prediction model for pit drip irrigation emitters based on BP and BP-PSO algorithms. The generalization ability of the models was evaluated through external validation. The performance of six different machine learning algorithms (CatBoost, LSBoost, MLP-SVM, MLP-DT, GR, MLR) in predicting the flow index of pit drip irrigation emitters was compared. The main conclusions are summarized as follows.

(1) The MSE and R^2^ for different numbers of neurons in the hidden layers were calculated and compared. It was determined that the optimal structure of the BP_1_ was 4–7-1, and the optimal structure of the BP_2_ was 4-4-9-1.

(2) The BP and BP-PSO can rapidly and accurately predict the flow index of pit drip irrigation emitters. The BP-PSO model had good prediction performance by avoiding getting trapped in local optima during the optimization process, which can achieve effective training and avoid large errors. The double-hidden-layer models outperform the single-hidden-layer models, with the BP_2_-PSO model having the most advantages and showing the best model performance.

(3) During the external validation process, the R^2^ value of the BP_2_ model was 0.9049, and the prediction errors were concentrated in the range of −0.005 to 0.005, demonstrating the best generalization performance. The other three models also exhibited good accuracy and generalization ability.

(4) Compared with CatBoost, LSBoost, MLP-SVM, MLP-DT, GR, MLR, the BP_2_-PSO model achieved the best prediction results in the training-testing process The BP_2_-PSO model had significant advantages in predicting the flow index of pit drip irrigation emitters.

## Data Availability

The data that support the findings of this study are available from the corresponding author upon reasonable request.
